# Global, regional, and national burden of gout among older adults (≥65) from 1990 to 2021 and projections for 2050

**DOI:** 10.3389/fpubh.2025.1540190

**Published:** 2025-06-16

**Authors:** Xiaoli Tang, Dan Deng, Qian Wu

**Affiliations:** ^1^Endocrinology and Metabolism, West China Hospital, Sichuan University, Chengdu, Sichuan, China; ^2^Division of Vascular Surgery, Department of General Surgery, West China Hospital, Sichuan University/West China School of Nursing, Sichuan University, Chengdu, Sichuan, China

**Keywords:** older adults, Global Burden of Disease, gout, prediction, risk factors

## Abstract

**Background:**

Gout, a common and treatable type of inflammatory arthritis, is caused by the buildup of monosodium urate crystals in the synovial fluid and other tissues. This study uses epidemiological modeling to analyze the global impact of gout among older adults (≥65) from 1990 to 2021, with projections extending to 2050, using the most recent data from the Global Burden of Disease (GBD) 2021.

**Method:**

Data from the GBD 2021 database were used to evaluate the global burden of gout in adults aged 65 and older from 1990 to 2021. The analysis considered factors such as age group (65+), gender, year (1990–2021), geographic classification [204 countries/regions, five socio-demographic index (SDI) regions, 21 GBD regions], and the SDI. Metrics assessed included incidence, prevalence, and disability-adjusted life years (DALYs), with their 95% uncertainty intervals (UI). All metrics were age-standardized using the GBD global standard population.

**Results:**

In 2021, the global age-standardized incidence rate of gout among older adults was 432.70 (95% UI: 263.28, 677.27) per 100,000 population. The age-standardized prevalence rate was 3,110.84 (95% UI: 2,092.83, 4,419.20) per 100,000, and the age-standardized DALYs were 90.90 (95% UI: 54.95, 139.13) per 100,000 person-years. There was an increasing trend in the incidence rate, prevalence, and DALYs as age increased among those 65 and older. The disease burden among older males was nearly twice that of females. Projections for 2050 show that the age-standardized incidence rate, prevalence rate, and DALYs are expected to increase to 524.99, 3,628.85, and 105.36 per 100,000 population, respectively. Metabolic risks have become the primary risk factor for gout.

**Discussion:**

Due to global population aging, our predictive model estimates that by 2050, the number of older adults with gout will increase by 8.5 million. The rise is particularly pronounced in high-SDI and high-income regions, highlighting the need for stronger prevention and management strategies in these areas. Early intervention in metabolic risk factors and improved early diagnosis and treatment are essential for effective gout management.

## 1 Introduction

Gout is a chronic inflammatory arthropathy driven by dysregulation of uric acid metabolism, characterized by persistent hyperuricemia (serum uric acid concentration >6.8 mg/dl) and an inflammatory response secondary to monosodium urate (MSU) crystal deposition (1). Hyperuricemia typically results from abnormalities in purine metabolism, impaired renal uric acid excretion, and dysregulated enzymatic activities, including overactivation of phosphoribosyl pyrophosphate synthetase, deficiency of hypoxanthine-guanine phosphoribosyltransferase, and increased catalytic activity of xanthine oxidase (2). Additionally, dysfunction of urate transporters in renal proximal tubules further compromises uric acid clearance (3). When serum uric acid levels surpass the solubility threshold, MSU crystals precipitate preferentially within the low-pH, low-temperature microenvironment of the joints, initiating a cascade of innate immune responses. Macrophage phagocytosis of MSU crystals triggers assembly of the NLRP3 inflammasome, culminating in caspase-1 activation, IL-1β maturation and release, and subsequent massive recruitment of neutrophils, leading to acute inflammation and tissue injury (4). During the chronic phase, persistent crystal deposition promotes tophus formation, characterized by imbalanced macrophage (M1/M2) polarization and fibrotic encapsulation, ultimately resulting in cartilage degradation, bone erosion, and irreversible joint deformities.

Beyond acute gouty arthritis and intense pain episodes, gout exerts systemic effects through sustained inflammation, contributing to multi-organ damage and posing a significant burden on patient health. With the accelerating global aging process, the incidence of gout among older adult is increasing annually. The global prevalence of gout is estimated to be 1%−6.8%, with rates ranging from 1 to 4% in Europe and 3.9% in the United States (5–7). According to the 2008 U.S. National Health and Nutrition Examination Survey, ~4.7 million older adult individuals in the country have gout (8). Moreover, a cohort study indicated that about 31.9% of gout patients were diagnosed at the age of 65 or older (9).

Studies have shown that the risk of developing gout increases significantly with age and is particularly prevalent in aging populations (10). This disease not only severely impacts individual physical and mental health and increases economic burdens but also poses significant challenges to global healthcare systems. According to the 2020 Global Burden of Disease (GBD) study, the global years lived with disability (YLDs) due to gout increased from 122,000 in 1990 to 239,000 in 2020 (11).

Currently, most studies on gout utilize data collected prior to 2019, focusing primarily on general populations or disease burden analyses limited to specific regions (9–16). However, there is a lack of sufficient evidence on the prevalence of gout and its global epidemiological trends specifically in older adult population. Therefore, a comprehensive assessment of the current global gout burden in older adult, along with medium- and long-term trend projections, is urgently needed to inform effective strategies.

This study utilized the latest 2021 GBD data and applied epidemiological models to systematically analyze the global burden of gout in older adult from 1990 to 2021 and projected trends for 2050. The study aims to uncover differences in the gout burden among older adult across gender, age groups, and regional distributions, providing scientific evidence for targeted interventions and resource allocation strategies.

## 2 Methods

### 2.1 Data sources

The GBD 2021 database (https://vizhub.healthdata.org/gbd-results/), which offers disease burden data for 371 diseases and 88 risk variables across 204 nations and territories worldwide, served as the sole source of statistics for this investigation. The database collects data and evaluates metrics using established procedures (38). The study focused on extracting gout-related data for individuals aged 65 years and older from 1990 to 2021 to assess its prevalence trends and disease burden.

The extracted data included the following dimensions: demographic characteristics (age group 65+, gender, years 1990–2021), geographic classifications (204 countries/territories globally, including WHO member states, five SDI regions, and 21 GBD regions), and the socio-demographic index (SDI), which measures socioeconomic context through indicators such as total fertility rate for individuals under 25, mean years of schooling for individuals aged 15 and above, and per capita lag-distributed income (39). Five SDI levels were applied to the 204 countries and territories: low, low-middle, middle, high-middle, and high. Within the 21 GBD regions, regional classifications were made according to geographic proximity and epidemiological similarity (40).

### 2.2 Analytical metrics

This study used measures including incidence, prevalence, and Disability-Adjusted Life Years (DALYs) and their associated 95% uncertainty intervals (UI) to analyze the disease burden of gout in people 65 and older. The age distribution of the GBD global standard population was used to compute age-standardized rates for incidence, prevalence, and DALYs.

### 2.3 Statistical methods

Statistical analyses of the global gout disease burden in older adults from 1990 to 2021 were conducted using Excel 2021 and R (version 4.3.3). After cleaning and organizing the collected data, statistical analyses and visualizations were performed using R packages such as dplyr, officer, and ggplot2. Statistical significance was established at a level of *P* < 0.05.

#### 2.3.1 Age-group analysis

Permission must be obtained for use of copyrighted material from other sources (including the web). Please note that it is compulsory to follow figure instructions.

The study population was split into seven age groups: 65–69, 70–74, 75–79, 80–84, 85–89, 90–94, and 95+ years old, in accordance with the age-grouping guidelines of GBD 2021. The dynamics of gout disease burden variations across these age groups and genders were investigated.

#### 2.3.2 Trend analysis

Percentage change (EAPC): R software was used to calculate and visualize the percentage change in gout disease burden across age groups, genders, regions, and countries from 1990 to 2021. The Estimated Annual Percentage Change (EAPC) was used to quantify trends in age-standardized rates. An EAPC with a 95% confidence interval lower bound >0 indicates an upward trend in age-standardized rates, whereas a lower bound ≤ 0 indicates a downward trend (41).

Joinpoint Regression Model: The Joinpoint regression model, which determines the Average Annual Percent Change (AAPC) and Annual Percent Change (APC) for particular periods, was used to study time patterns from 1990 to 2021. If the 95% confidence interval includes 0, the trend is considered stable. An AAPC or APC significantly > or < 0 indicates an upward or downward trend, respectively, for the relevant metrics (42). Model fitting and AAPC/APC calculations were conducted using Joinpoint software (version 4.9.0.0) with visualizations produced in R.

#### 2.3.3 Correlation analysis

The association between age-standardized gout rates and the SDI was assessed using Pearson correlation analysis. Furthermore, the percentages of gout attributable to kidney disease, metabolic hazards, and high body mass index were examined.

### 2.4 Decomposition and forecast analysis

Decomposition analysis: while controlling for other variables, decomposition techniques were used to measure the separate contributions of aging, population expansion, and epidemiological changes to the burden of gout (43).

Age-period-cohort analysis: the dynamic impact of age, period, and cohort characteristics on gout were evaluated using the Age-Period-Cohort (APC) model. To examine the impact of variables on gout incidence and mortality risk, the APC model, which is based on a Poisson distribution, breaks them down into three dimensions: age, period, and cohort (44).

Bayesian age-period-cohort model (BAPC): building on the APC model, the BAPC model incorporates Bayesian Markov Chain Monte Carlo algorithms to address parameter estimation challenges caused by the linear relationship among the three factors. This model was used to predict the burden of gout from 2022 to 2050 (45).

## 3 Results

### 3.1 Global burden of gout in older adults

In 2021, the age-standardized incidence rate of gout among older adults was 432.70 (95% UI: 263.28, 677.27) per 100,000 population, the age-standardized prevalence rate was 3,110.84 (95% UI: 2,092.83, 4,419.20) per 100,000 population, and the age-standardized DALYs were 90.90 (95% UI: 54.95, 139.13) per 100,000 person-years. This indicates that 3,318,171.28 (95% UI: 2,018,433.81, 5,197,653.84) new gout cases occurred in 2021, including 2,207,869.69 (95% UI: 1,339,816.28, 3,459,256.11) males and 1,110,301.60 (95% UI: 678,416.45, 1,741,293.44) females. The total number of gout cases in 2021 was 23,730,400.86 (95% UI: 15,953,641.14, 33,717,972.23), of which 16,184,603.23 (95% UI: 10,879,440.36, 22,961,992.04) were males and 7,545,797.63 (95% UI: 5,014,123.94, 10,792,915.18) were females. Gout resulted in 695,181.04 (95% UI: 419,889.66, 1,063,310.19) DALYs in 2021, including 476,926.92 (95% UI: 288,335.10, 728,546.18) DALYs among males and 218,254.11 (95% UI: 130,848.00, 334,754.44) DALYs among females. From 1990 to 2021, all disease burden metrics for gout in older adults showed positive EAPC values, indicating a general increasing trend in the global disease burden of gout in this population (see Table 1).

**Table 1:** Age-standardized gout burden findings for 21 GBD regions, five SDI regions, and the general population worldwide.

**Location**	**Incidence**			**Prevalence**			**DALYs**		
	**1990 (per 100,000 population, 95% UI)**	**2021 (per 100,000 population, 95% UI)**	**EAPCs (95% CI)**	**1990 (per 100,000 population, 95% UI)**	**2021 (per 100,000 population, 95% UI)**	**EAPCs (95% CI)**	**1990 (per 100,000 population, 95% UI)**	**2021 (per 100,000 population, 95% UI)**	**EAPCs (95% CI)**
Global	355.83 (215.85, 557.29)	432.70 (263.28, 677.27)	0.76 (−0.06, 1.58)	2,454.32 (1,624.89, 3,519.53)	3,110.84 (2,092.83, 4,419.20)	1.01 (0.14, 1.90)	71.94 (42.87, 111.17)	90.90 (54.95, 139.13)	0.37 (−0.54, 1.28)
**SDI**									
High SDI	378.41 (225.24, 608.57)	471.11 (281.95, 750.64)	0.97 (0.24, 1.71)	3,159.65 (2,100.04, 4,488.78)	4,528.67 (3,133.57, 6,270.79)	1.61 (0.69, 2.55)	92.78 (55.80, 144.09)	131.85 (82.21, 200.16)	0.22 (−0.72, 1.17)
High-middle SDI	345.99 (210.53, 540.07)	451.78 (274.43, 707.95)	0.89 (0.02, 1.77)	2,255.66 (1,484.93, 3,257.27)	2,966.36 (1,950.86, 4,278.31)	0.96 (0.03, 1.90)	66.46 (39.20, 103.39)	87.48 (51.76, 136.75)	0.35 (−0.55, 1.25)
Middle SDI	360.75 (220.73, 557.48)	442.03 (270.38, 682.62)	0.79 (−0.06, 1.64)	2,101.07 (1,376.14, 3,044.23)	2,644.07 (1,725.03, 3,838.55)	0.92 (0.08, 1.77)	61.91 (36.48, 96.79)	77.71 (46.09, 120.65)	0.26 (−0.70, 1.22)
Low-middle SDI	321.77 (197.28, 496.85)	348.43 (213.59, 540.01)	0.32 (−0.59, 1.23)	1,852.83 (1,206.57, 2,683.50)	2,026.56 (1,316.54, 2,937.43)	0.35 (−0.54, 1.25)	53.46 (31.40, 84.38)	58.66 (34.40, 92.13)	1.00 (0.12, 1.90)
Low SDI	343.51 (210.72, 532.66)	358.45 (220.58, 556.81)	0.19 (−0.75, 1.13)	1,984.30 (1,291.12, 2,880.34)	2,070.89 (1,344.39, 3,009.71)	0.19 (−0.74, 1.13)	57.12 (33.71, 89.44)	59.94 (34.90, 93.23)	1.58 (0.64, 2.52)
**GBD 21 regions**									
Andean Latin America	165.22 (100.83, 257.16)	208.53 (125.91, 324.01)	0.82 (−0.18, 1.83)	908.25 (591.44, 1,330.45)	1,172.71 (762.57, 1,707.16)	0.93 (−0.05, 1.93)	27.09 (14.87, 44.24)	34.91 (19.06, 56.85)	0.16 (−0.82, 1.15)
Australasia	563.44 (339.11, 891.16)	693.52 (406.80, 1,120.74)	0.60 (−0.13, 1.34)	5,569.48 (3,740.21, 7,735.77)	7,641.08 (5,170.47, 10,784.04)	1.01 (0.03, 2.00)	163.12 (99.16, 250.96)	224.58 (134.72, 349.07)	0.52 (−0.50, 1.55)
Caribbean	151.32 (91.88, 234.97)	184.27 (112.19, 287.12)	0.68 (−0.12, 1.49)	809.61 (525.01, 1,180.70)	1,007.25 (654.78, 1,468.57)	0.76 (0.01, 1.51)	24.25 (13.78, 39.88)	29.77 (16.79, 46.74)	0.78 (0.09, 1.48)
Central Asia	290.11 (176.37, 452.20)	339.34 (205.72, 533.84)	0.42 (−0.51, 1.37)	1,719.53 (1,106.97, 2,509.10)	2,063.45 (1,335.54, 3,007.75)	0.50 (−0.44, 1.45)	50.86 (29.29, 80.49)	60.96 (35.29, 96.48)	0.34 (−0.72, 1.42)
Central Europe	264.98 (162.29, 410.88)	296.02 (180.64, 459.73)	0.34 (−0.53, 1.23)	1,512.81 (982.34, 2,198.34)	1,720.87 (1,116.64, 2,498.66)	0.40 (−0.48, 1.29)	43.99 (25.57, 69.24)	50.14 (29.17, 79.32)	0.61 (−0.36, 1.60)
Central Latin America	101.61 (61.21, 158.71)	121.36 (73.38, 189.56)	0.83 (0.13, 1.53)	561.68 (359.86, 827.16)	673.75 (432.88, 991.68)	0.84 (0.21, 1.46)	16.77 (9.72, 26.89)	20.08 (11.50, 31.59)	0.71 (−0.30, 1.73)
Central Sub-Saharan Africa	365.90 (222.50, 575.36)	353.91 (216.40, 556.89)	0.05 (−0.90, 1.01)	2,172.34 (1,387.08, 3,199.21)	2,092.25 (1,354.91, 3,055.86)	0.03 (−0.92, 0.99)	62.42 (35.40, 99.63)	60.53 (33.95, 96.29)	0.39 (−0.53, 1.32)
East Asia	443.06 (271.39, 685.28)	573.83 (350.75, 892.18)	1.07 (0.27, 1.87)	2,610.07 (1,704.20, 3,784.65)	3,491.11 (2,279.91, 5,080.42)	1.23 (0.45, 2.03)	77.49 (45.53, 121.15)	103.22 (60.69, 161.37)	0.26 (−0.59, 1.11)
Eastern Europe	297.18 (181.66, 461.06)	349.86 (214.25, 541.33)	0.41 (−0.57, 1.40)	1,694.24 (1,097.89, 2,464.42)	2,028.67 (1,313.84, 2,940.99)	0.46 (−0.54, 1.47)	49.24 (28.50, 77.50)	58.99 (34.49, 92.80)	0.58 (−0.30, 1.48)
Eastern Sub-Saharan Africa	377.11 (230.79, 584.64)	387.83 (238.38, 602.36)	0.20 (−0.75, 1.16)	2,188.59 (1,418.47, 3,183.78)	2,266.76 (1,470.09, 3,292.41)	0.22 (−0.73, 1.18)	63.35 (36.75, 99.04)	66.03 (38.86, 103.40)	0.92 (0.06, 1.78)
High-income Asia Pacific	398.45 (238.83, 639.96)	437.00 (261.63, 702.85)	0.12 (−0.64, 0.88)	2,889.32 (1,878.15, 4,213.99)	3,336.33 (2,182.66, 4,826.12)	0.32 (−0.57, 1.23)	85.42 (50.61, 133.36)	99.34 (58.87, 155.15)	0.97 (0.03, 1.92)
High-income North America	476.98 (279.69, 771.62)	634.34 (378.78, 1,012.96)	1.79 (1.13, 2.45)	4,201.96 (2,783.23, 5,986.53)	7,271.47 (5,202.45, 9,829.73)	2.99 (2.08, 3.91)	122.63 (73.37, 191.62)	208.12 (133.99, 307.70)	2.92 (2.01, 3.85)
North Africa and Middle East	355.96 (216.65, 553.84)	420.18 (255.89, 658.11)	0.53 (−0.36, 1.44)	2,112.88 (1,373.38, 3,069.19)	2,546.17 (1,646.21, 3,730.94)	0.61 (−0.26, 1.50)	61.79 (35.97, 96.20)	73.92 (43.08, 116.37)	0.48 (−0.52, 1.49)
Oceania	486.65 (297.95, 754.36)	522.77 (317.27, 812.39)	0.21 (−0.66, 1.09)	2,942.78 (1,916.54, 4,294.82)	3,216.84 (2,092.74, 4,640.71)	0.27 (−0.57, 1.11)	85.90 (49.88, 135.19)	93.41 (53.57, 146.18)	1.22 (0.42, 2.04)
South Asia	329.57 (202.24, 509.62)	356.55 (219.13, 551.97)	0.33 (−0.58, 1.25)	1,883.44 (1,224.20, 2,723.50)	2,047.89 (1,333.06, 2,969.42)	0.36 (−0.54, 1.27)	53.66 (31.20, 84.64)	58.76 (34.22, 92.01)	0.06 (−0.89, 1.03)
Southeast Asia	385.01 (237.13, 594.42)	456.87 (277.69, 711.87)	0.59 (−0.38, 1.58)	2,270.74 (1,481.90, 3,276.63)	2,778.85 (1,818.19, 4,037.46)	0.70 (−0.30, 1.71)	66.54 (39.39, 103.87)	81.53 (47.85, 128.27)	0.81 (0.22, 1.40)
Southern Latin America	402.43 (238.92, 657.41)	458.99 (269.98, 732.68)	0.31 (−0.45, 1.08)	3,476.85 (2,277.59, 4,980.36)	4,298.43 (2,853.71, 6,099.71)	0.62 (−0.34, 1.60)	103.16 (59.90, 163.51)	126.94 (75.99, 197.25)	0.41 (−0.47, 1.29)
Southern Sub-Saharan Africa	418.91 (256.50, 648.83)	448.65 (274.53, 699.58)	0.33 (−0.70, 1.37)	2,466.51 (1,606.15, 3,575.51)	2,665.44 (1,730.56, 3,854.83)	0.37 (−0.68, 1.44)	72.05 (41.78, 113.02)	77.00 (45.54, 121.74)	0.49 (−0.46, 1.45)
Tropical Latin America	155.19 (94.67, 240.51)	191.35 (116.70, 297.12)	0.74 (−0.04, 1.53)	831.74 (542.46, 1,218.06)	1,041.87 (677.83, 1,519.37)	0.79 (0.07, 1.53)	24.24 (14.19, 38.58)	30.33 (17.59, 48.12)	0.72 (0.01, 1.44)
Western Europe	283.58 (168.57, 456.33)	309.61 (183.73, 498.26)	0.21 (−0.56, 0.99)	2,537.37 (1,690.64, 3,589.81)	3,020.17 (2,020.11, 4,262.92)	0.52 (−0.49, 1.54)	74.88 (44.78, 115.55)	89.16 (53.65, 139.01)	1.00 (0.02, 2.00)
Western Sub-Saharan Africa	350.76 (214.37, 545.22)	363.90 (222.61, 562.95)	0.09 (−0.88, 1.06)	2,035.13 (1,323.26, 2,968.72)	2,136.03 (1,394.34, 3,110.66)	0.13 (−0.84, 1.11)	59.18 (34.28, 92.73)	62.48 (36.75, 97.02)	0.92 (−0.04, 1.88)

### 3.2 Regional burden of gout in older adults

Among the five SDI regions, the High SDI region had the highest age-standardized incidence rate [471.11 (95% UI: 281.95, 750.64)], age-standardized prevalence rate [4,528.67 (95% UI: 3,133.57, 6,270.79)], and age-standardized DALYs [131.85 (95% UI: 82.21,200.16)]. Conversely, the low-middle SDI region had the lowest age-standardized incidence rate [348.43 (95% UI: 213.59, 540.01)], age-standardized prevalence rate [2,026.56 (95% UI: 1,316.54, 2,937.43)], and age-standardized DALYs [58.66 (95% UI: 34.40, 92.13)]. From a temporal perspective, all gout burden metrics increased across SDI regions. The High SDI region showed the most significant increases in incidence rate [EAPC = 0.97 (95% UI: 0.24, 1.71)] and prevalence rate [EAPC = 1.61 (95% UI: 0.69, 2.55)], while the Low SDI region exhibited the most significant increase in DALYs [EAPC = 1.58 (95% UI: 0.64, 2.52); see Table 1].

Among the 21 GBD regions, Australasia had the highest age-standardized incidence rate (693.52, 95% UI: 406.80, 1,120.74), age-standardized prevalence rate (7,641.08, 95% UI: 5,170.47, 10,784.04), and age-standardized DALYs (224.58, 95% UI: 134.72, 349.07) in 2021. In contrast, Central Latin America had the lowest age-standardized incidence rate (121.36, 95% UI: 73.38, 189.56), age-standardized prevalence rate (673.75, 95% UI: 432.88, 991.68), and age-standardized DALYs (20.08, 95% UI: 11.50, 31.59). From a temporal perspective, the disease burden increased across all GBD regions. High-income North America exhibited the most severe increases, with the incidence rate (EAPC = 1.79, 95% UI: 1.13, 2.45), prevalence rate (EAPC = 2.99, 95% UI: 2.08, 3.91), and DALYs (EAPC = 2.92, 95% UI: 2.01, 3.85) showing the greatest growth (see Table 1).

### 3.3 National and regional burden of gout in older adults

**Figure 1:**
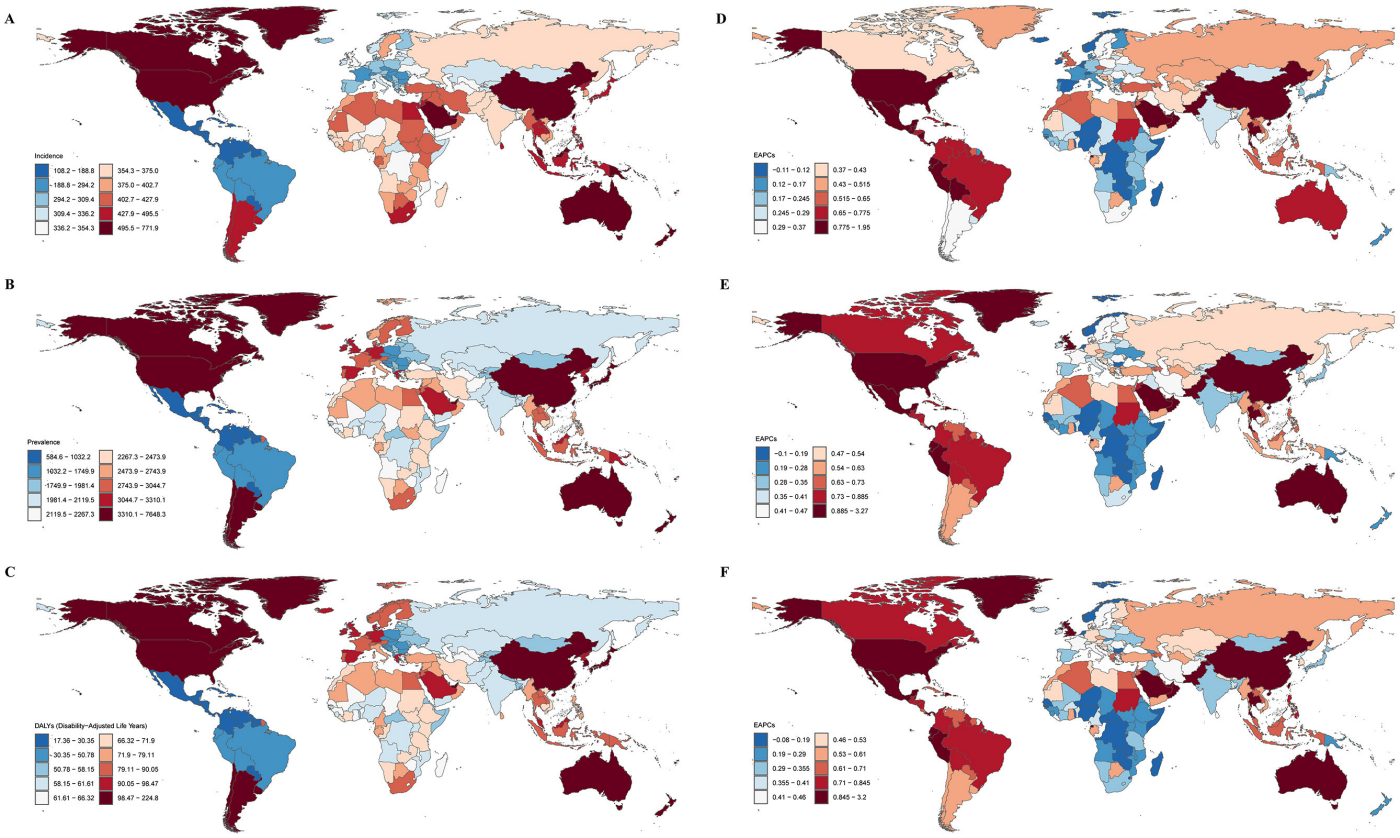
The burden of gout in 204 countries and regions globally in 2021. **(A)** Age-standardized incidence rate; **(B)** age-standardized prevalence rate; **(C)** age-standardized DALYs; **(D)** EAPC for incidence rate; **(E)** EAPC for prevalence rate; **(F)** EAPC for DALYs.

With an age-standardized incidence rate of 771.87 (95% UI: 446.87, 1,250.88) per 100,000 population, New Zealand had the highest rate of gout among older persons in 2021. Australia had the highest age-standardized DALYs (224.77, 95% UI: 134.77, 347.77) per 100,000 person-years and the highest age-standardized prevalence rate (7,648.30, 95% UI: 5,202.84, 10,791.44) per 100,000 population. Guatemala, on the other hand, had the lowest DALYs (17.36, 95% UI: 8.51, 29.81), prevalence rate (584.64, 95% UI: 373.13, 852.27), and age-standardized incidence rate (108.18, 95% UI: 65.44, 170.21; see Figures 1A–C). The prevalence of gout illness declined in Nigeria, the Democratic Republic of the Congo, and Somalia, according to longitudinal trend research. Furthermore, there were declining trends in Zimbabwe's age-standardized DALYs and the Netherlands' age-standardized incidence rate. However, it is noteworthy that the decreasing trends in these countries were not significant, as the 95% confidence intervals (CIs) of the EAPCs included 0. In contrast, the disease burden of gout in older adults increased in all other countries and regions (see Figures 1D–F). Among these, the United States of America exhibited the most pronounced upward trends in the age-standardized incidence rate (EAPC = 1.95, 95% UI: 1.29, 2.62), prevalence rate (EAPC = 3.27, 95% UI: 2.35, 4.20), and DALYs (EAPC = 3.20, 95% UI: 2.27, 4.13). Detailed disease burden data for 204 countries and regions are provided in Supplementary material 1.

### 3.4 Age-sex-time association analysis of gout in older adults

**Figure 2:**
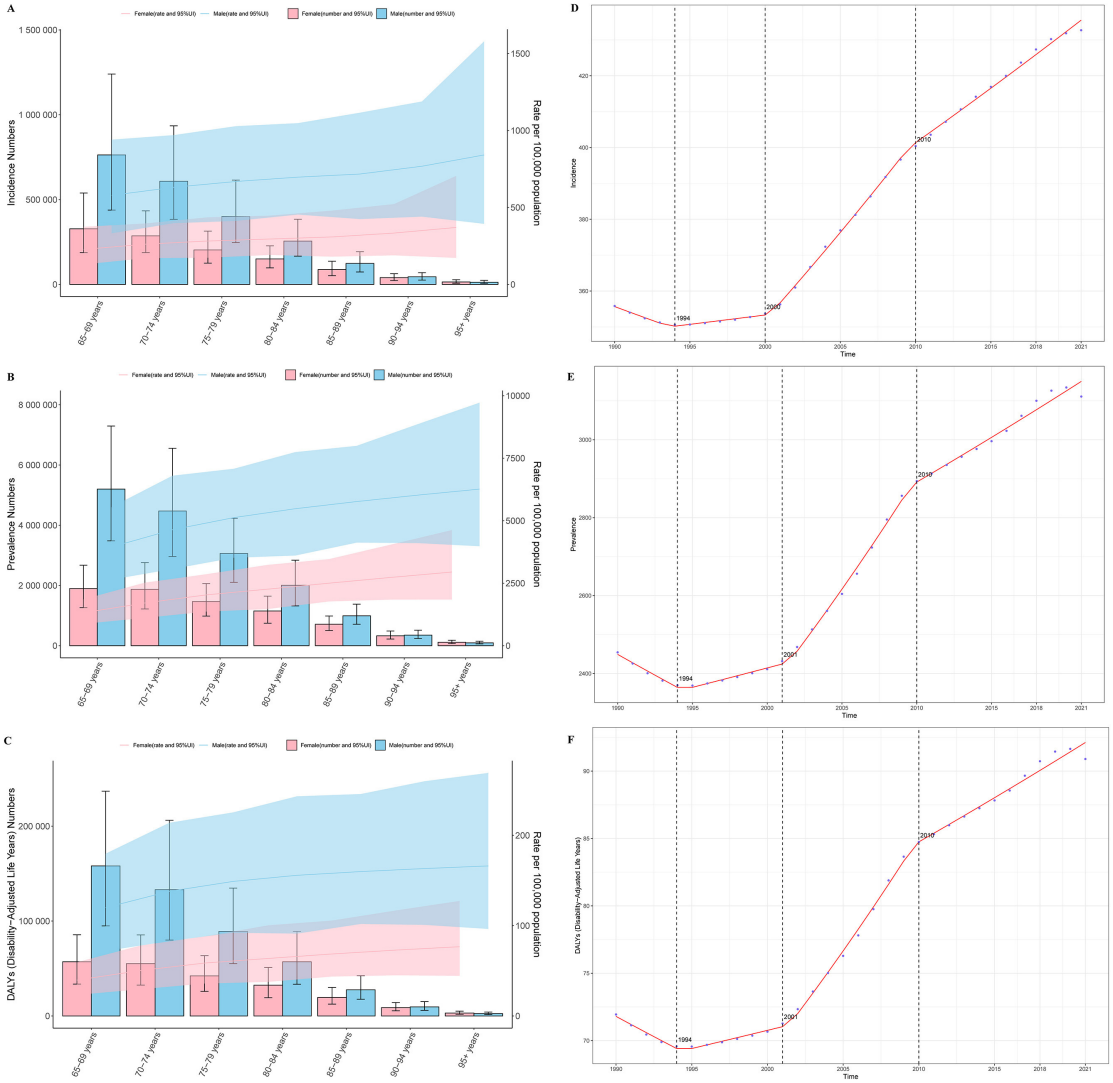
Trends in the age- and sex-specific burden of gout and Joinpoint regression analysis results. **(A)** Incidence; **(B)** prevalence; **(C)** DALYs; **(D)** joinpoint analysis of incidence; **(E)** joinpoint analysis of prevalence; **(F)** joinpoint analysis of DALYs.

Age-sex association analysis revealed that, in 2021, the incidence rate, prevalence rate, and DALYs of gout increased with age among individuals aged 65 and older. However, this increase was not statistically significant. Additionally, the disease burden among older males was nearly twice as high as that among females (see Figures 2A–C).

Age-time association analysis demonstrated that, globally, the age-standardized incidence rate, prevalence rate, and DALYs of gout across different age groups remained relatively stable from 1990 to 2021, showing no significant changes over time. However, the disease burden of gout increased with age, with older individuals bearing a higher burden (see Supplementary Figures 1–3).

Sex-time association analysis indicated that, globally, the age-standardized incidence rate, prevalence rate, and DALYs of gout among older adults remained relatively stable for both males and females from 1990 to 2021, without significant changes over time. Notably, the disease burden among males was approximately twice that of females (see Supplementary Figures 4–6).

### 3.5 Trends in the burden of gout in older adults

Joinpoint regression analysis revealed an overall increasing trend in the incidence, prevalence, and DALYs of gout among older adults globally from 1990 to 2021. Specifically, the AAPC for incidence was 2.575 (95% CI: 2.528, 2.621), for prevalence was 22.545 (95% CI: 21.740, 23.350), and for DALYs was 0.654 (95% CI: 0.630, 0.678). Significant changes in these three disease burden metrics were observed in the years 1994, 2001 (2000 for incidence), and 2010 (see Figures 2D–F). Since 2010, the upward trend in gout burden has slightly slowed but remains significantly increasing. The slope changes for each curve segment are detailed in Supplementary material 2.

### 3.6 Association between gout burden and SDI in older adults

**Figure 3:**
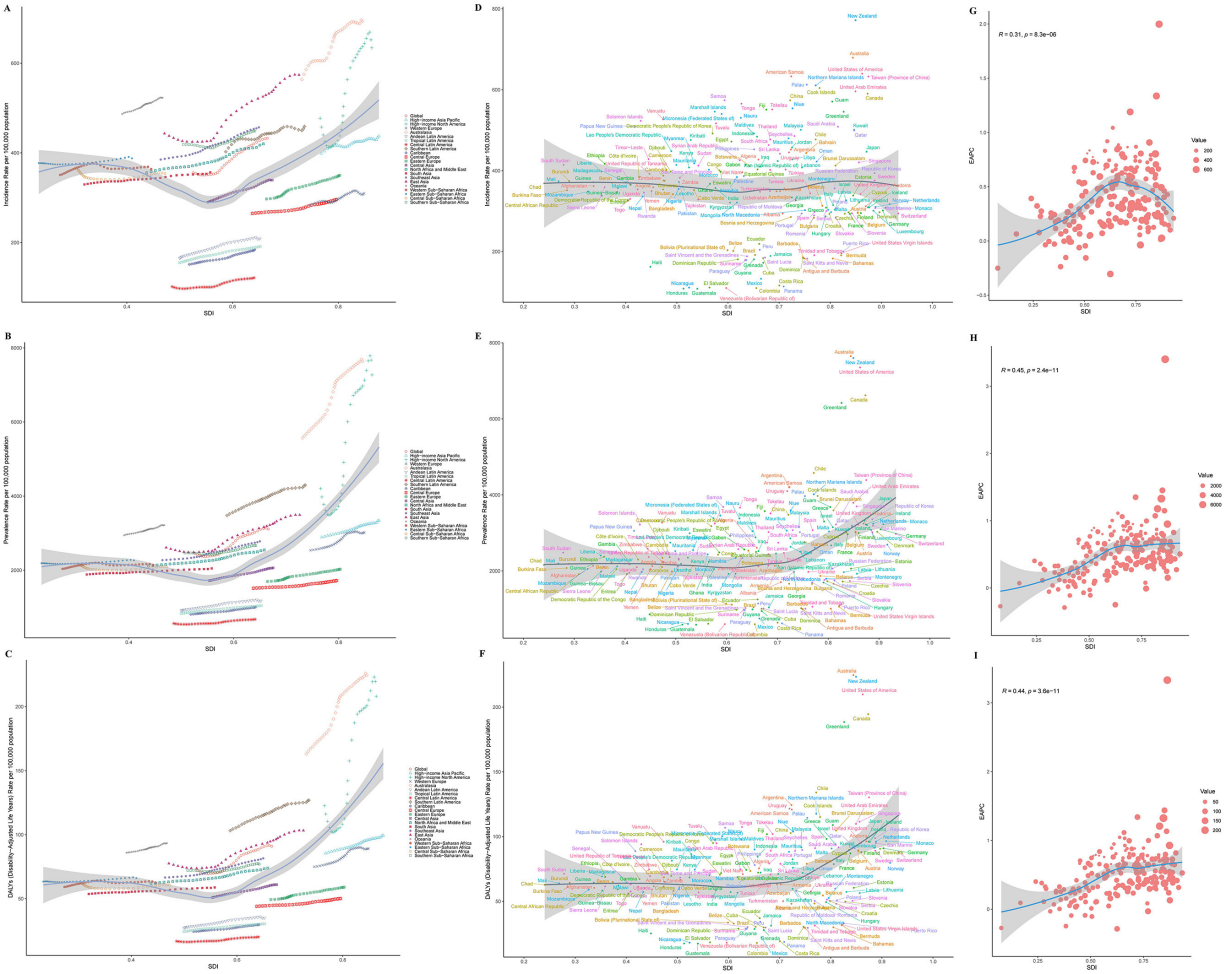
SDI analysis results. **(A)** Incidence in 21 regions; **(B)** prevalence in 21 regions; **(C)** DALYs in 21 regions; **(D)** incidence in 204 countries; **(E)** prevalence in 204 countries; **(F)** DALYs in 204 countries; **(G)** EAPC of incidence; **(H)** EAPC of prevalence; **(I)** EAPC of DALYs.

Globally and across the 21 GBD regions, the relationship between SDI and the age-standardized incidence rate, prevalence rate, and DALYs of gout displayed a non-linear trend. Specifically, as SDI increased, the burden of gout initially decreased slowly, reaching its lowest point around an SDI value of 0.55. Beyond this point, the burden of gout began to increase slowly with higher SDI values. Among the 21 GBD regions, Australasia experienced the fastest growth in disease burden (see Figures 3A–C). Across 204 countries, there was no significant correlation between gout incidence and SDI (*p* = 1.508e-01). However, gout prevalence (*p* = 9.542e-06) and DALYs (*p* = 5.197e-06) increased significantly with higher SDI values. Additionally, the EAPC for prevalence and DALYs also increased with SDI, indicating that in higher SDI countries, the burden of gout is becoming more severe (see Figures 3D–I).

### 3.7 Results of age-period-cohort analysis of gout burden in older adults

**Figure 4:**
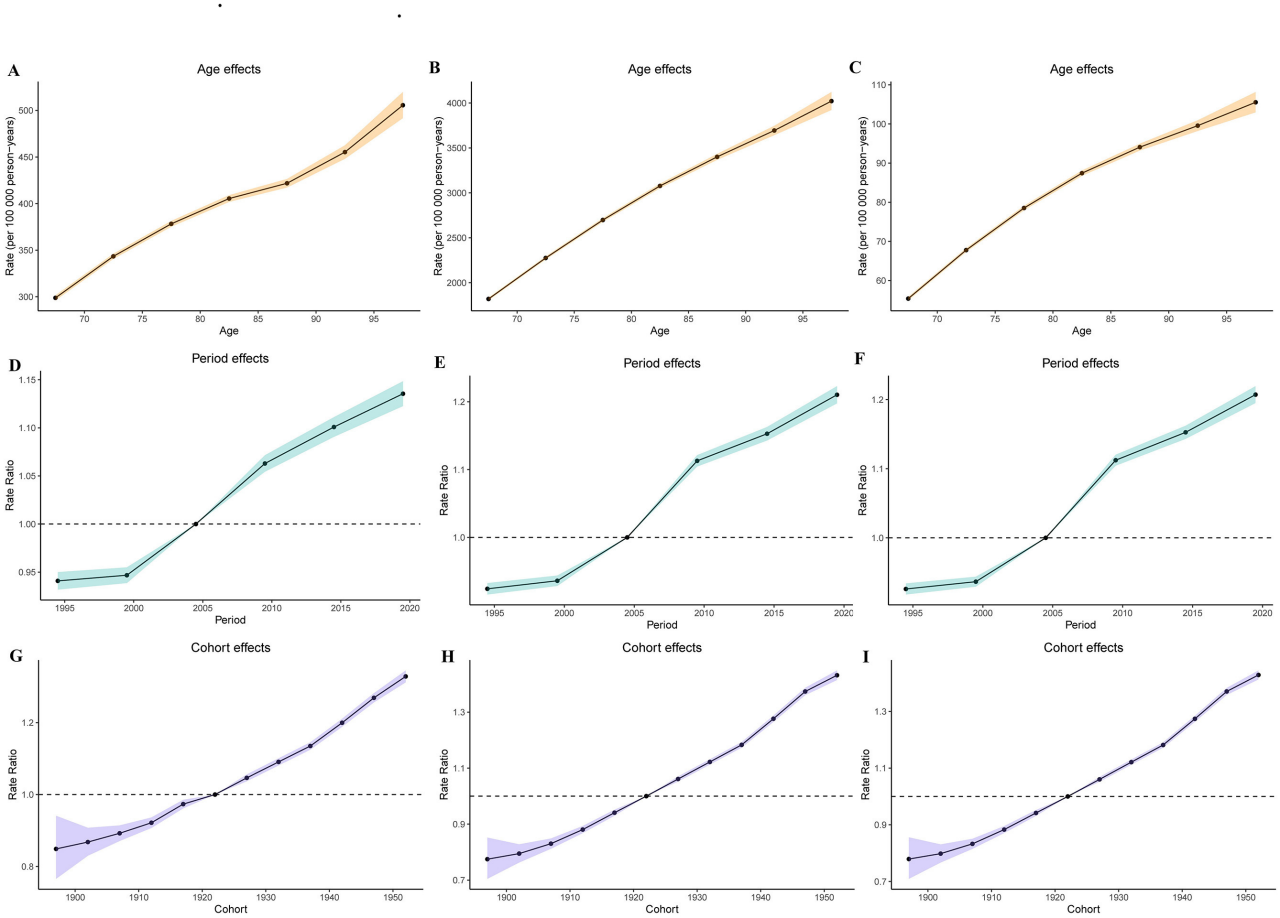
Age-period-cohort analysis results. **(A)** Age effect for incidence; **(B)** age effect for prevalence; **(C)** age effect for DALYs; **(D)** period effect for incidence; **(E)** period effect for prevalence; **(F)** period effect for DALYs; **(G)** cohort effect for incidence; **(H)** cohort effect for prevalence; **(I)** Cohort effect for DALYs.

The age-period-cohort analysis for the incidence, prevalence, and DALYs of gout in older adults demonstrated similar trends. The age effect analysis revealed a significant upward trend in gout disease burden with increasing age (see Figures 4A–C). The period effect analysis showed a gradual increase in the burden of gout over time from 1990 to 2021 (see Figures 4D–F). The cohort effect analysis indicated a significantly higher gout burden among later-born cohorts compared to earlier-born cohorts (see Figures 4G–I).

### 3.8 Results of decomposition analysis of gout burden in older adults

**Figure 5:**
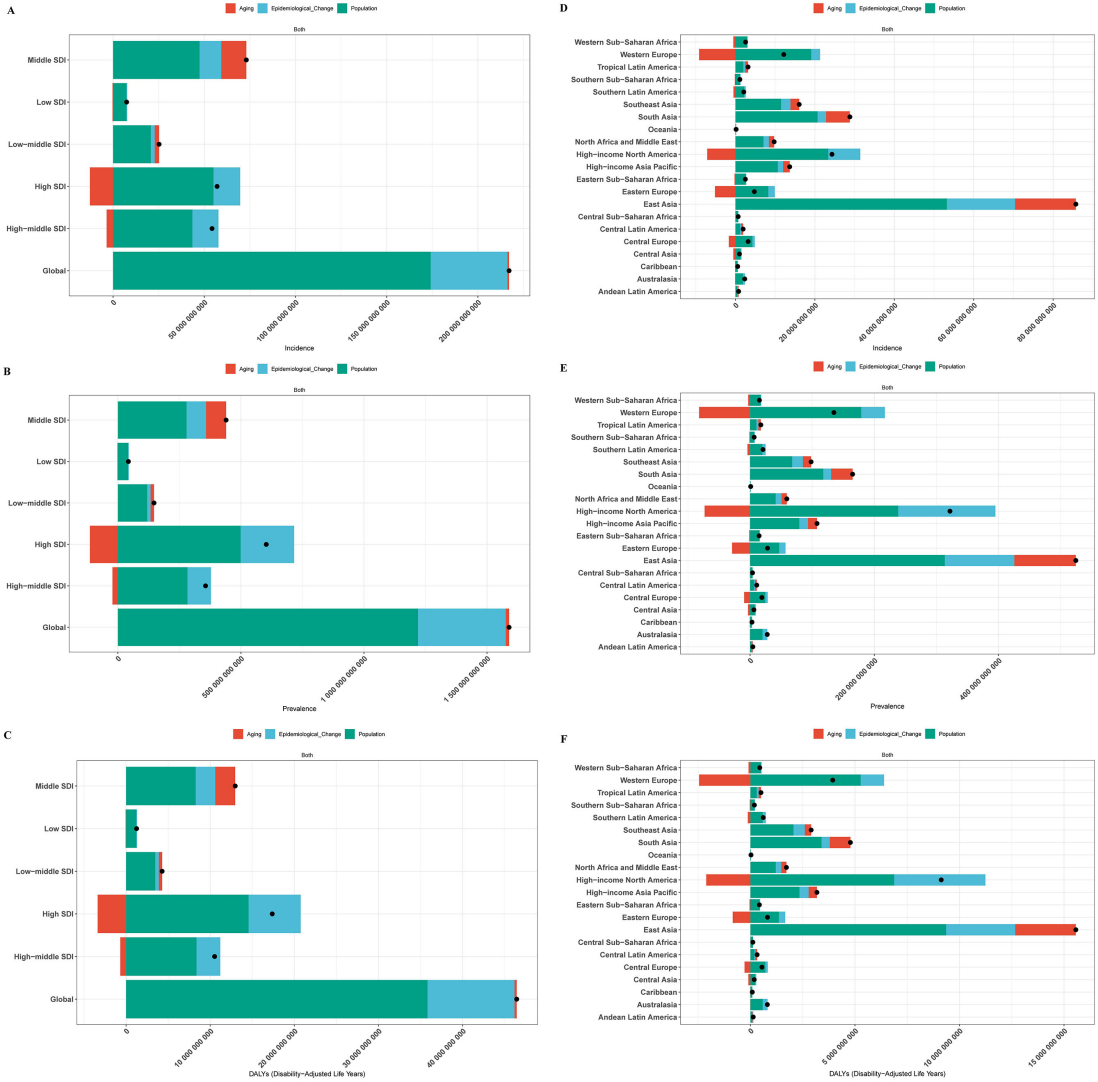
Decomposition analysis results. **(A)** Incidence globally and in five SDI regions; **(B)** prevalence globally and in five SDI regions; **(C)** DALYs globally and in five SDI regions; **(D)** incidence in 21 GBD regions; **(E)** prevalence in 21 GBD regions; **(F)** DALYs in 21 GBD regions.

Consistent trends were found by the decomposition analysis in the contributions of epidemiological changes, aging, and population increase to the worldwide gout burden, as well as in the five SDI areas and the 21 GBD regions. In particular, aging significantly reduced the burden of gout in High SDI and High-Middle SDI regions, as well as in Western Europe, High-Income North America, and Eastern Europe. However, in other regions, the burden of gout increased due to the combined effects of population growth, aging, and epidemiological changes. The primary cause of the increase in the burden of gout among these was population growth (see Figure 5).

### 3.9 Results of predictive analysis of gout burden in older adults

**Figure 6:**
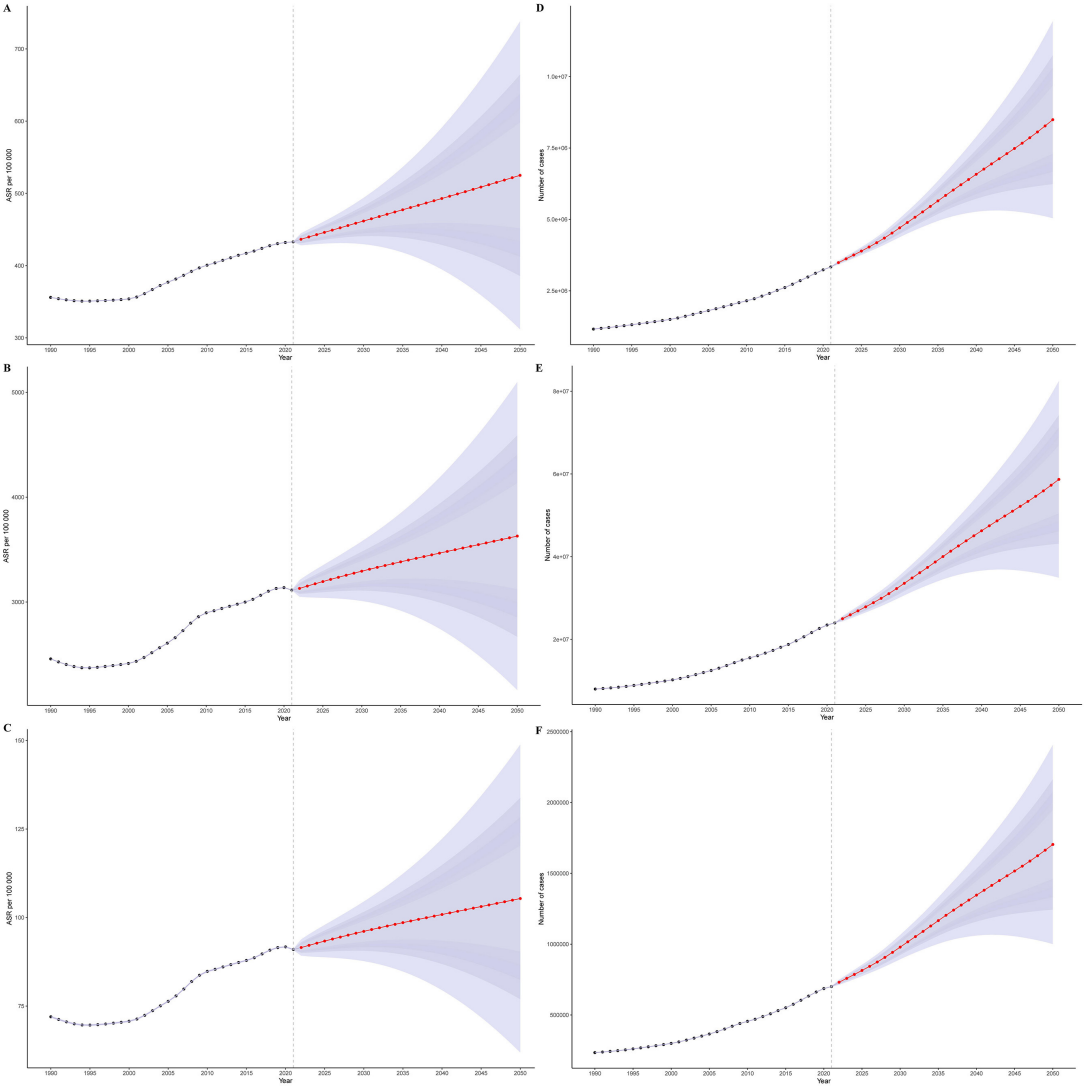
Predictive analysis results. **(A)** Age-standardized incidence rate; **(B)** age-standardized prevalence rate; **(C)** age-standardized DALYs; **(D)** actual values of incidence; **(E)** actual values of prevalence; **(F)** actual values of DALYs.

Predictive analysis indicates that the global burden of gout among older adults will continue to rise from 2022 to 2050 (see Figure 6). By 2050, the age-standardized incidence rate, prevalence rate, and DALYs of gout in older adults are projected to increase to 524.99 (95% CI: 311.84, 738.13) per 100,000 population, 3,628.85 (95% CI: 2,159.85, 5,097.85) per 100,000 population, and 105.36 (95% CI: 61.88, 148.84) per 100,000 person-years, respectively (see Supplementary material 3). This suggests that by 2050, there will be 8,490,224.04 (95% CI: 5,043,207.67, 11,937,240.41) new gout cases among individuals aged 65 and older. The total number of gout patients in this age group will reach 58,686,803.59 (95% CI: 34,929,674.77, 82,443,932.41), resulting in a loss of 1,703,873.011 (95% CI: 1,000,669.949, 2,407,076.073) life years (see Supplementary material 4). Furthermore, our predictive analysis across different age groups showed that the disease burden of gout exhibits a significant upward trend across all age groups. The older the age group, the more pronounced the upward trend (see Supplementary Figures 7–9).

### 3.10 Risk factors for DALYs

**Figure 7:**
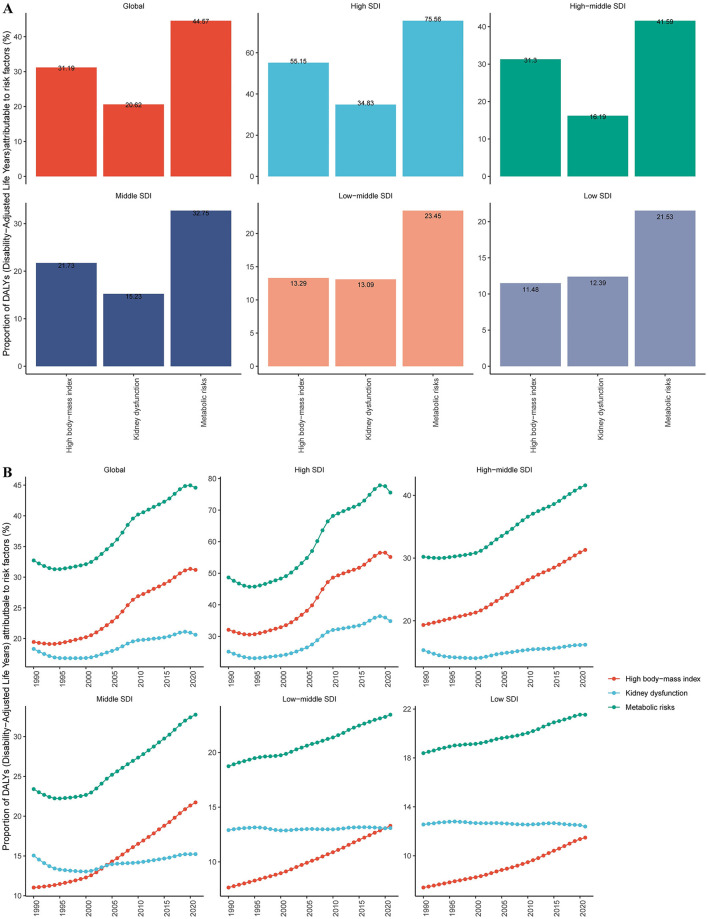
Global and five SDI regions' risk factors for gout DALYs. **(A)** Risk factors for gout in 2021. **(B)** Temporal trends of gout risk factors.

Currently, only the risk factors contributing to gout-related DALYs have been reported. Three risk factors contribute to DALYs among gout patients: high body-mass index, kidney dysfunction, and metabolic risks. Globally, across the five SDI regions, and within the 21 GBD regions, metabolic risks are the primary risk factor for gout-related DALYs (see Figure 7A and Supplementary Figure 10). Over time, the gout disease burden attributable to various risk factors has shown an upward trend, with the increase in metabolic risks being the most prominent (see Figure 7B and Supplementary Figure 11).

## 4 Discussion

This study conducted a systematic analysis of the global burden and temporal trends of gout among individuals aged 65 and older from 2019 to 2021, based on data from GBD 2021. The results showed that by 2021, the age-standardized incidence rate, prevalence, and DALYs for gout in older adults had increased by 21.6%, 26.7%, and 26.4%, respectively, compared to 1990. This upward trend is consistent with the findings of Han et al. (17), who analyzed the global gout burden based on GBD 1990–2019 data. However, due to differences in the study periods and populations, the results are not directly comparable, and only a general alignment in trend can be observed.

The study further found that the burden of gout increases significantly with age. Projections indicate that by 2050, the global number of older adult individuals affected by gout will approach 58 million. It is noteworthy that the rising trend in gout burden is not solely driven by population aging; multiple factors have also played critical roles.

Studies suggest that seasonal variations may influence the frequency of gout flares through environmental temperature and humidity changes. Low temperatures in winter may promote urate crystal precipitation, while dehydration during summer may elevate serum uric acid levels, both contributing to acute attacks (18).

Our study found that the burden of gout in older adult men was significantly higher than in women, with men experiencing nearly twice the disease burden compared to women. This finding is consistent with the results of the GBD 2021 Gout Collaborators (19), and may be closely related to both physiological mechanisms and social behavior differences. First, sex-based differences in uric acid metabolism likely account for much of this disparity. A retrospective analysis indicated that men generally have higher serum uric acid levels (20). In women, estrogen exerts a protective effect by promoting uric acid excretion via the urine, thereby reducing the risk of gout (21, 22). A cross-sectional study further showed that women have an increased risk of developing gout after menopause (23), likely due to the gradual loss of estrogen-mediated protection during this period (24). Second, social behavior differences may also contribute significantly to the gout burden. Alcohol consumption is recognized as a major trigger for gout flares (25). Several studies have demonstrated that men tend to consume more alcohol than women, thereby increasing their risk of developing gout (26–28). Moreover, dietary patterns are well-established modifiable risk factors for gout. Men are more likely to consume purine-rich foods such as red meat and seafood, leading to elevated uric acid levels and an increased risk of gout (29–31). It is also worth noting that sexual orientation may indirectly influence gout risk through associated lifestyle factors. Among men who have sex with men (MSM), some studies have observed higher rates of alcohol dependence and antiretroviral therapy (e.g., protease inhibitors) use, both of which may interfere with uric acid metabolism (32). However, direct epidemiological evidence linking sexual orientation itself to gout remains limited, as existing studies primarily focus on behavioral patterns rather than sexual identity. Additionally, medication use can impact gout risk. Diuretics inhibit OAT1/OAT3-mediated uric acid secretion, cyclosporine impairs ABCG2 function, and obesity and insulin resistance exacerbate urate retention by upregulating URAT1 expression (1).

In terms of geographical distribution, gout has shown a marked upward trend in regions with a high SDI and in high-income countries. The relationship between SDI and the burden of gout is non-linear: the burden is lowest in middle-SDI regions, while it rises significantly in high-SDI countries. This may be associated with the “Westernization” of lifestyles—for example, dietary habits in high-income countries (such as high-purine and high-calorie diets) and sedentary behaviors have contributed to an increased risk of metabolic diseases such as obesity and diabetes (33, 34). In contrast, in low-SDI regions, limited access to healthcare and insufficient health management may lead to underestimation or underdiagnosis of the disease.

Additionally, we looked at the high-risk factors that contribute to gout and found that the main risk factors for gout-related DALYs were metabolic problems, kidney dysfunction, and a high body mass index. Among these, the impact of metabolic risks was particularly significant and exhibited an upward trend. This indicates that gout is not only a joint disease but also a component of metabolic syndrome. These results align with the findings of previous studies conducted by other researchers (35–37).

Predictive analysis indicates that by 2050, the incidence, prevalence, and DALYs of gout among older adults will further increase, with a significant rise in the global number of gout patients and the overall disease burden. The burden is expected to grow particularly among the oldest age groups. In response to this trend, it is imperative to implement multi-level public health strategies, including: (1) establishing early screening programs targeting high-risk older adult populations; (2) promoting community-based health management and public education to enhance awareness and self-management of gout; (3) strengthening dietary interventions and weight control at the primary care level; and (4) encouraging interdisciplinary collaboration in comprehensive prevention and management, involving fields such as nutrition, endocrinology, and geriatric medicine.

In conclusion, this study comprehensively reveals the epidemiological characteristics and trends in the burden of gout among older adults worldwide, providing valuable evidence for future disease management and health policy development. With the intensifying population aging and the prevalence of metabolic diseases, strengthening multidisciplinary collaboration and addressing gout from the perspectives of prevention, management, and policy intervention will be crucial in effectively tackling the increasingly severe burden of gout.

## Data Availability

Publicly available datasets were analyzed in this study. This data can be found here: GBD 2021, https://ghdx.healthdata.org/gbd-2021.
