# Supplementary figures and images for: Global, regional, and national burden of gout among older adults (≥65) from 1990 to 2021 and projections for 2050
Source: Front Public Health. 2025 Jun 16;13:1540190. doi: 10.3389/fpubh.2025.1540190 (PMC12206735; doi:10.3389/fpubh.2025.1540190)

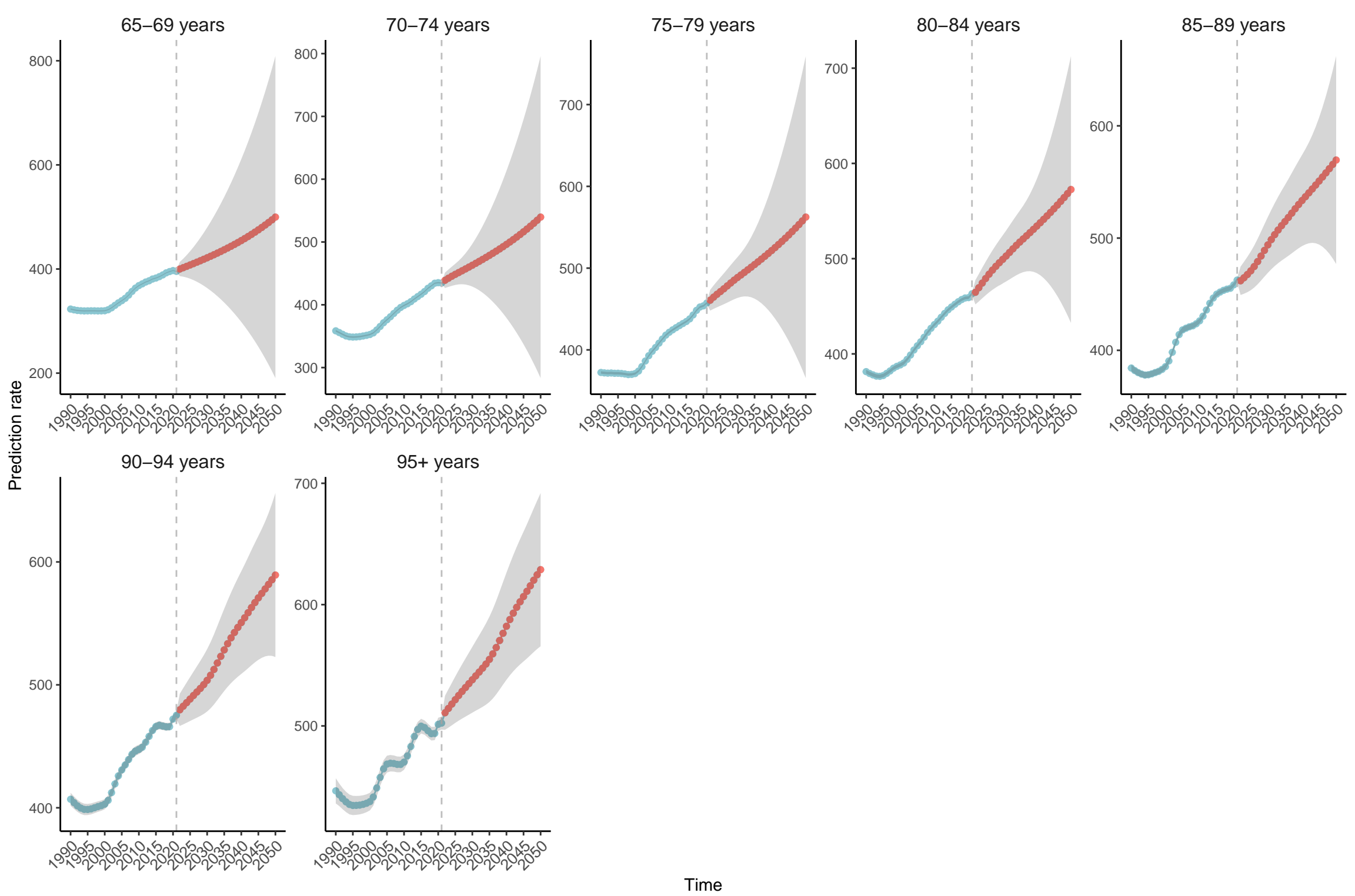

Supplement: Supplementary file 1 [file Data_Sheet_1.zip › Supplementary documents/Supplementary Figure 7Prevalence at different ages 2050.pdf]

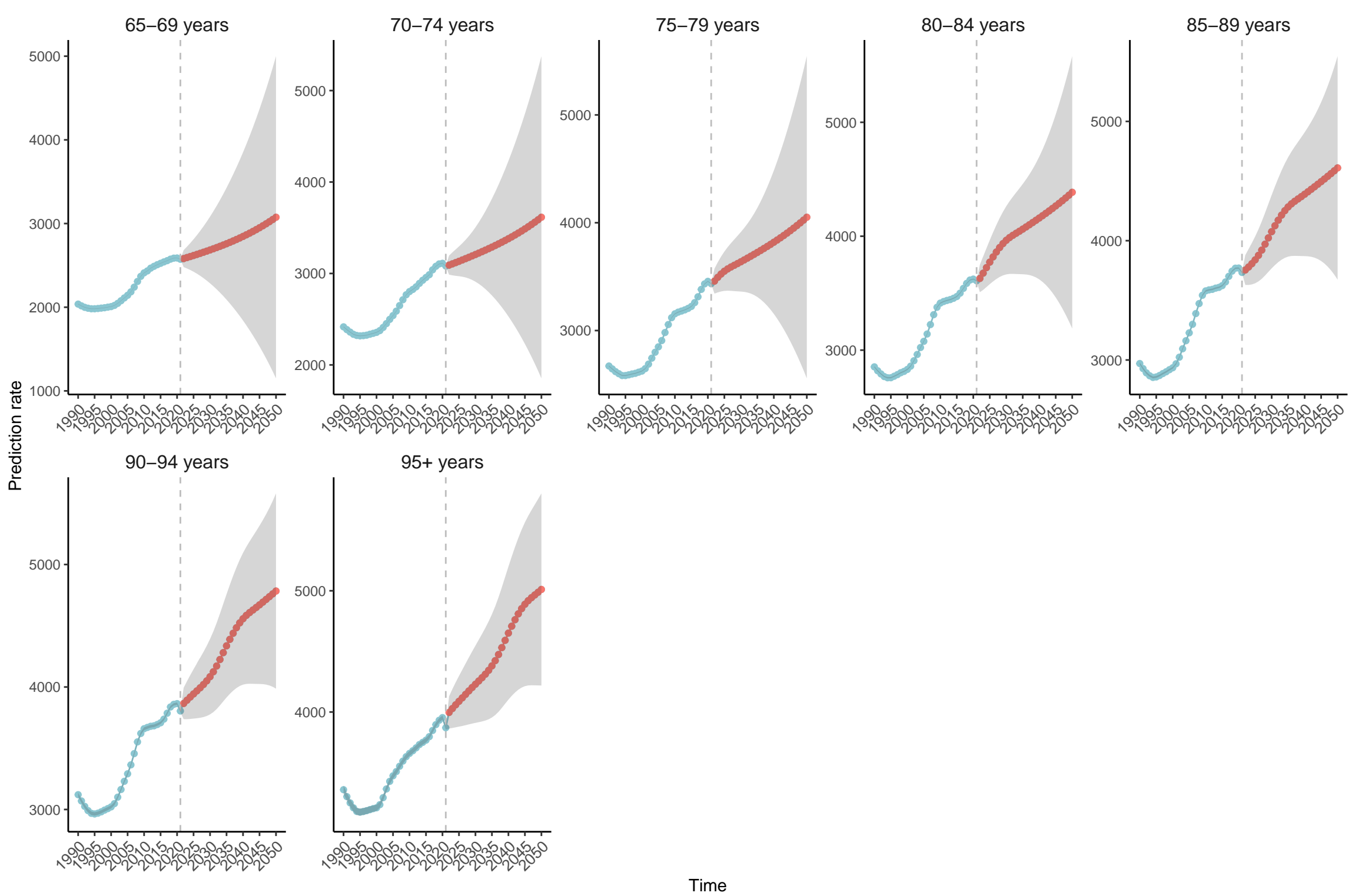

Supplement: Supplementary file 1 [file Data_Sheet_1.zip › Supplementary documents/Supplementary Figure 8∩╝ÜPrevalence in different age groups 2050.pdf]

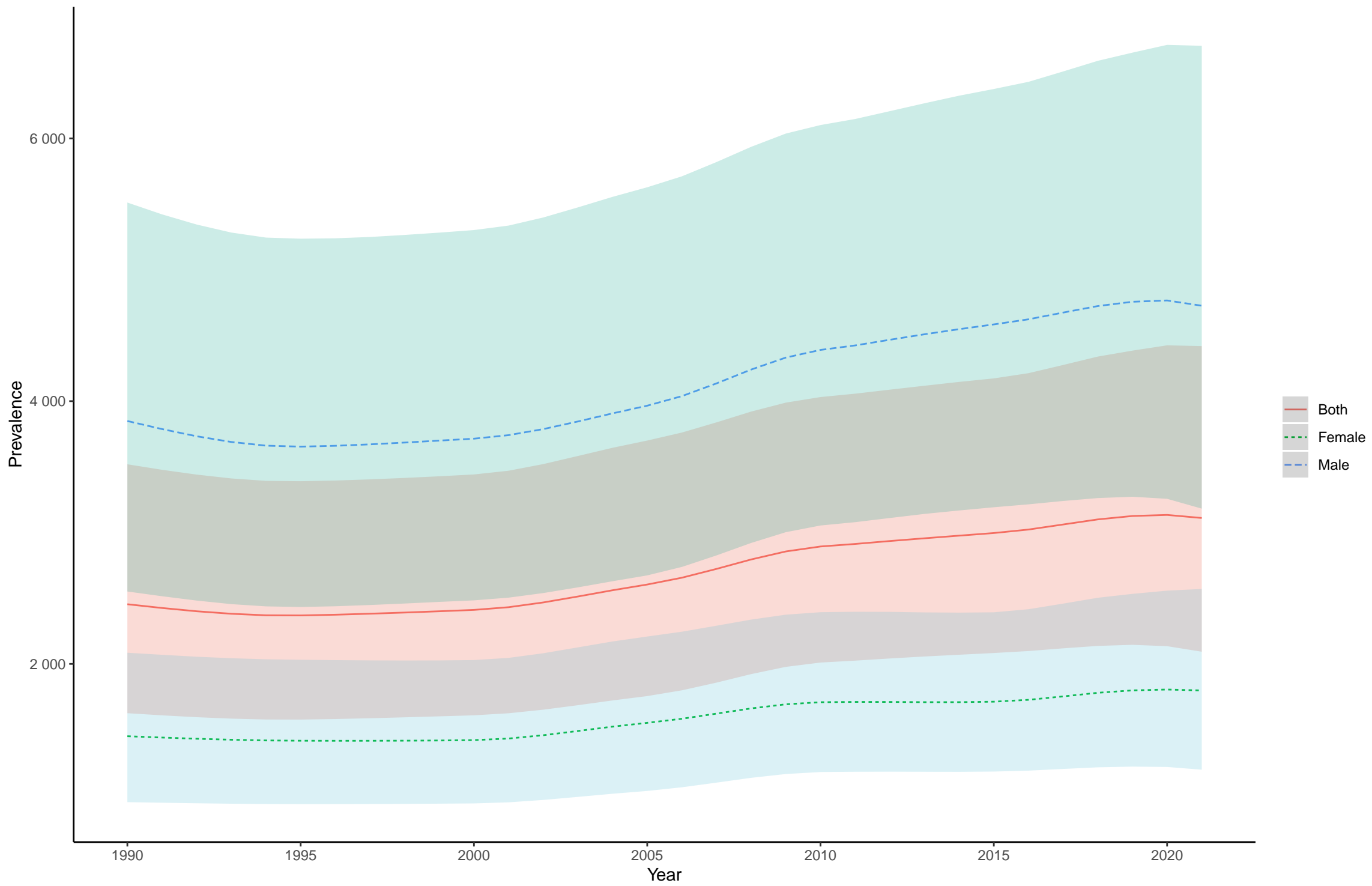

Supplement: Supplementary file 1 [file Data_Sheet_1.zip › Supplementary documents/Supplementary Figure 5∩╝Üprevalence-Trends in gender-specific prevalence rates over time.pdf]

DALYs (Disability-Adjusted Life Years)

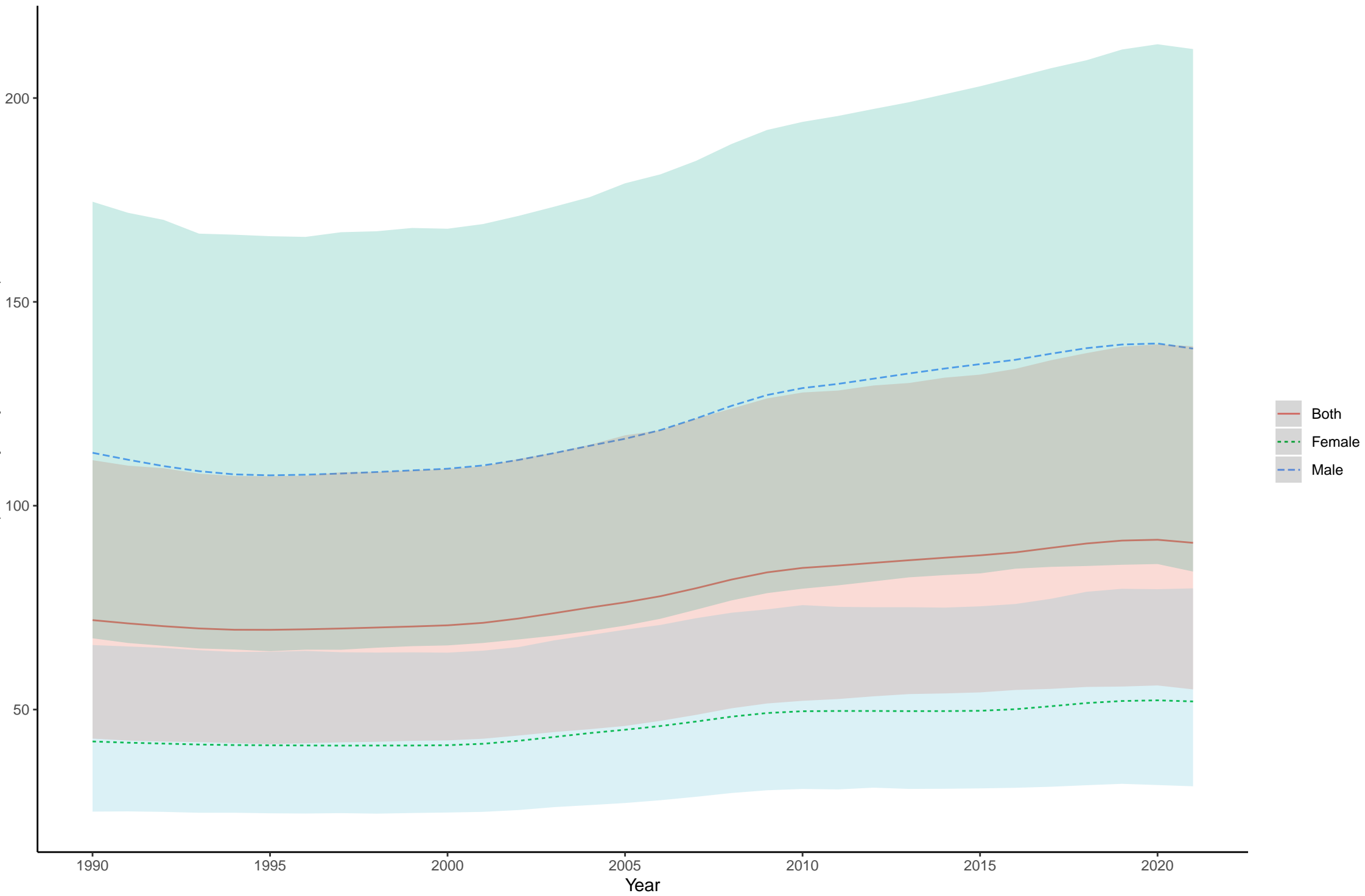

Supplement: Supplementary file 1 [file Data_Sheet_1.zip › Supplementary documents/Supplementary Figure 6∩╝ÜDAYLs-Trends in DAYLs of different sexes over time.pdf]

Global

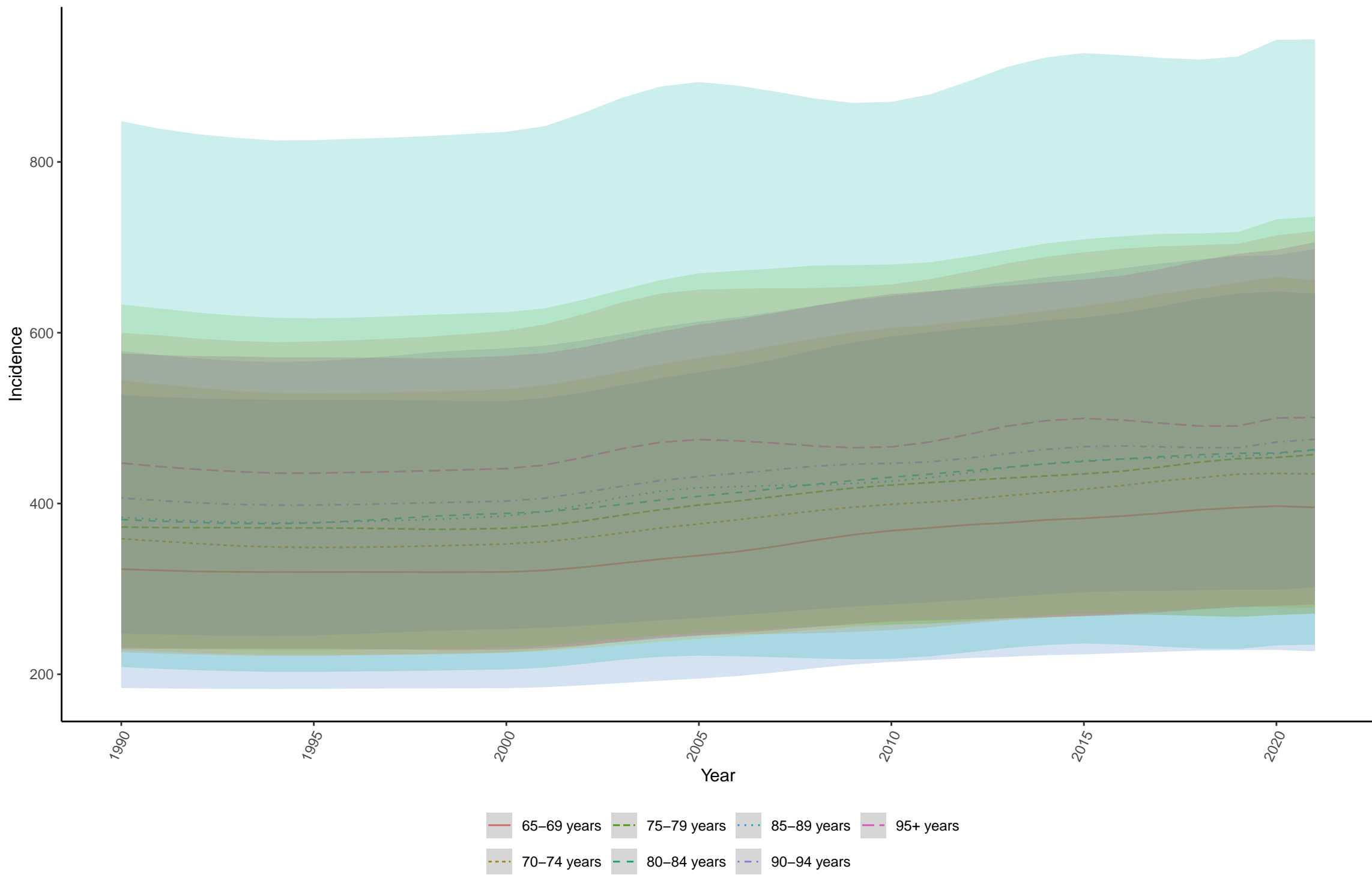

Supplement: Supplementary file 1 [file Data_Sheet_1.zip › Supplementary documents/Supplementary Figure 1∩╝ÜIncidence-Trends in prevalence over time in different age groups.pdf]

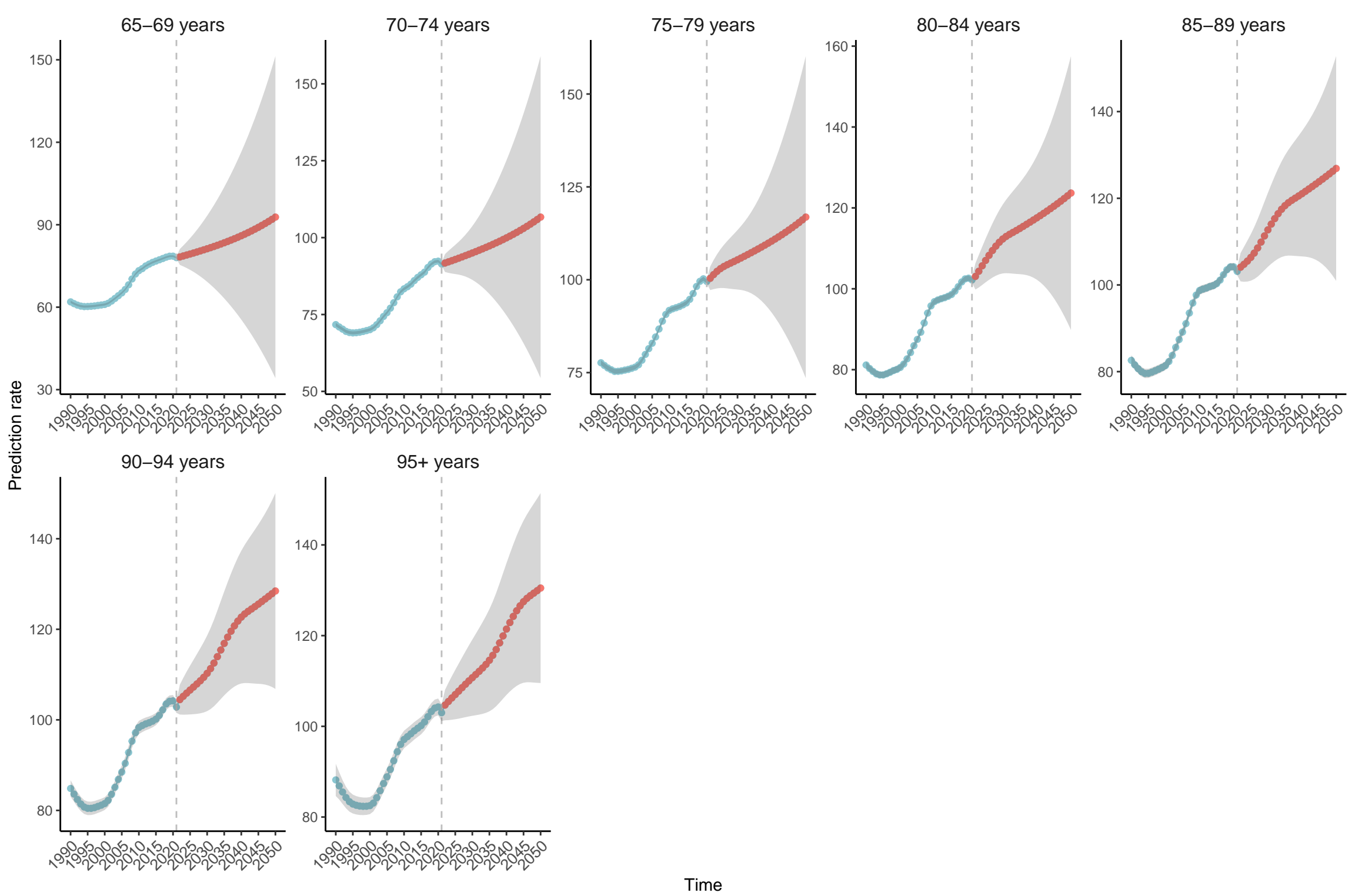

Supplement: Supplementary file 1 [file Data_Sheet_1.zip › Supplementary documents/Supplementary Figure 9DALYs2050 for different age groups.pdf]

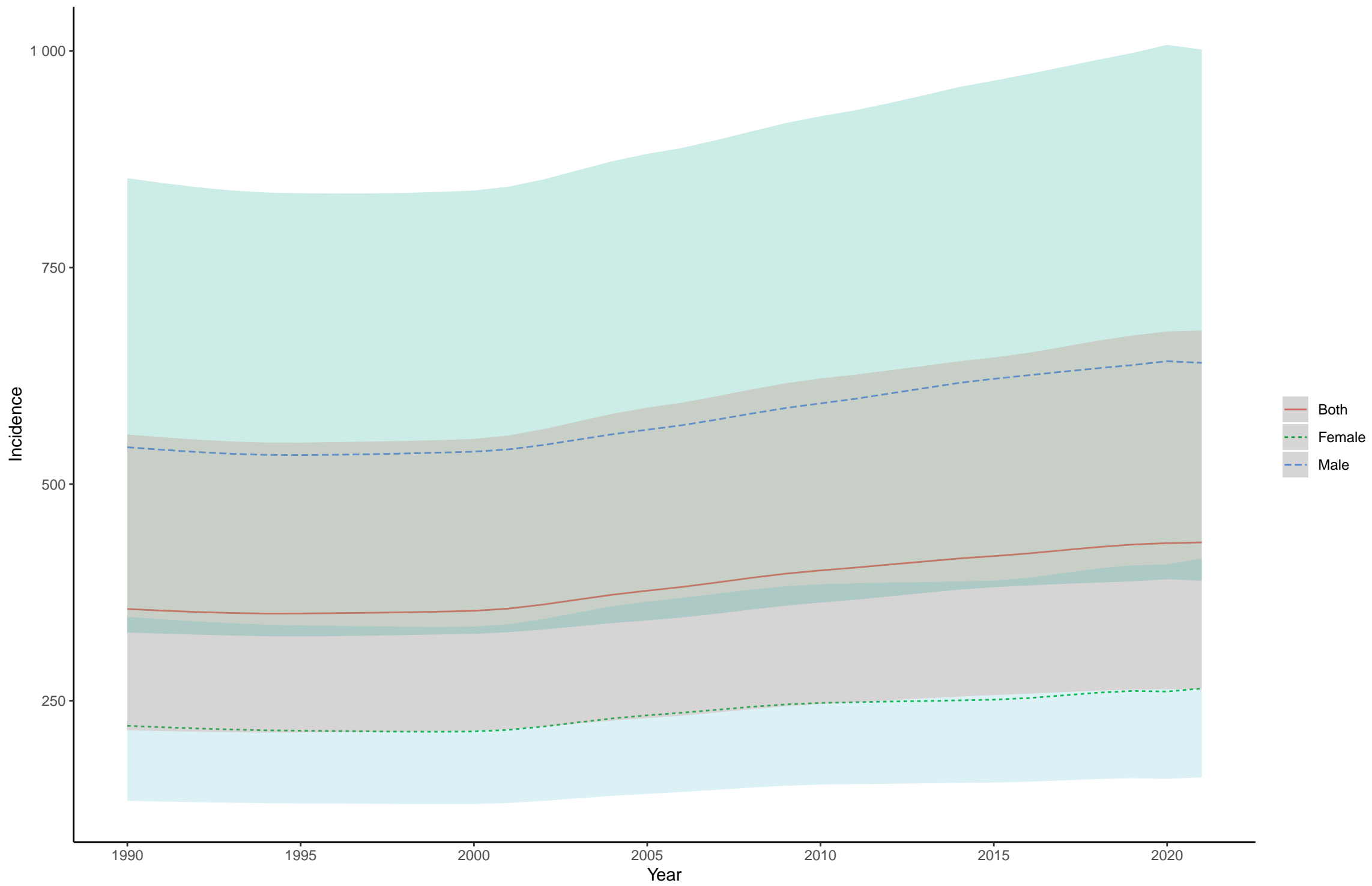

Supplement: Supplementary file 1 [file Data_Sheet_1.zip › Supplementary documents/Supplementary Figure 4∩╝ÜIncidence-Trends in the incidence of standardization over time by sex.pdf]

DALYs (Disability-Adjusted Life Years)

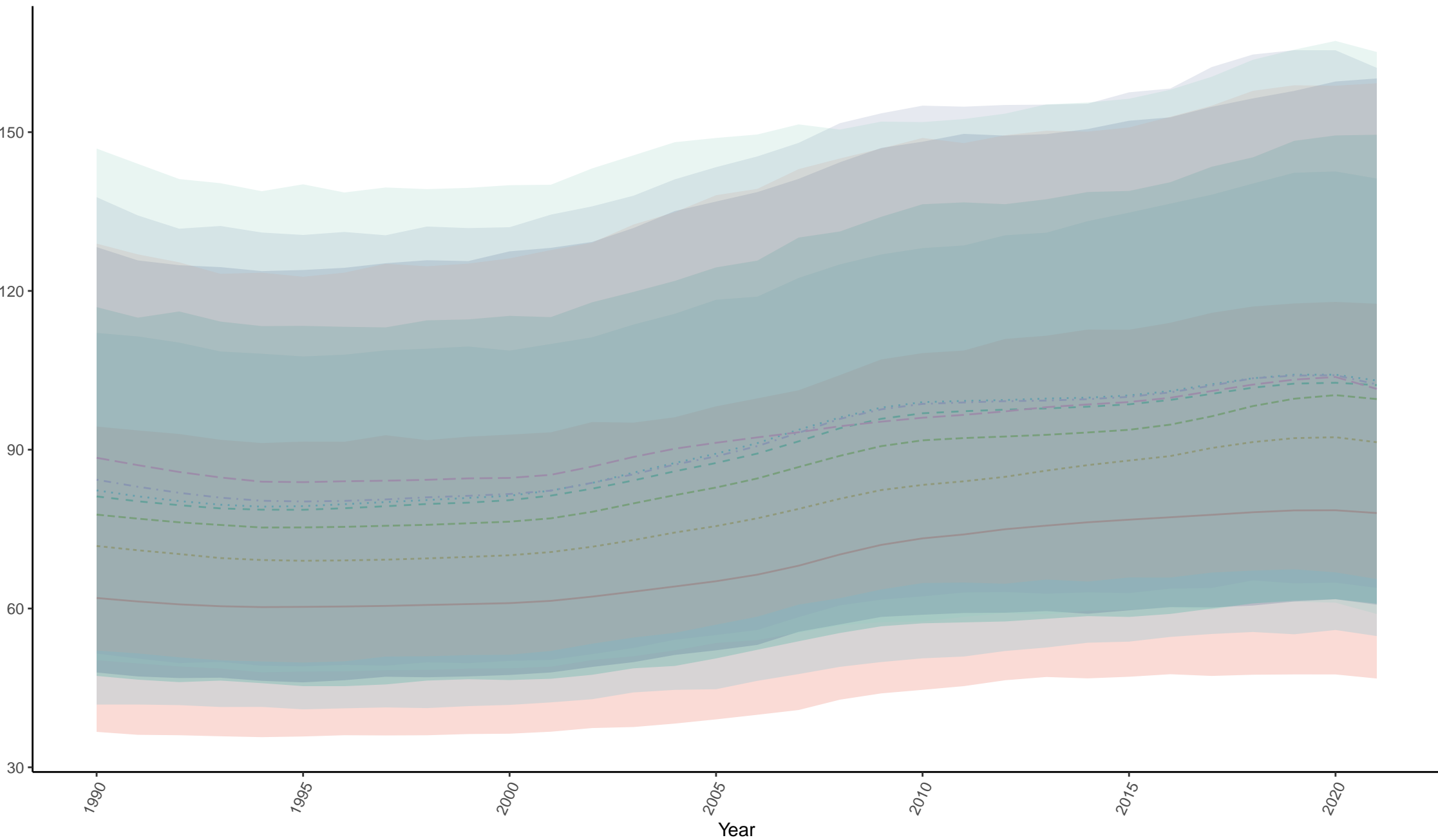

Supplement: Supplementary file 1 [file Data_Sheet_1.zip › Supplementary documents/Supplementary Figure 3Trends in DALYs over time for different age groups of DAYLs.pdf]

Global

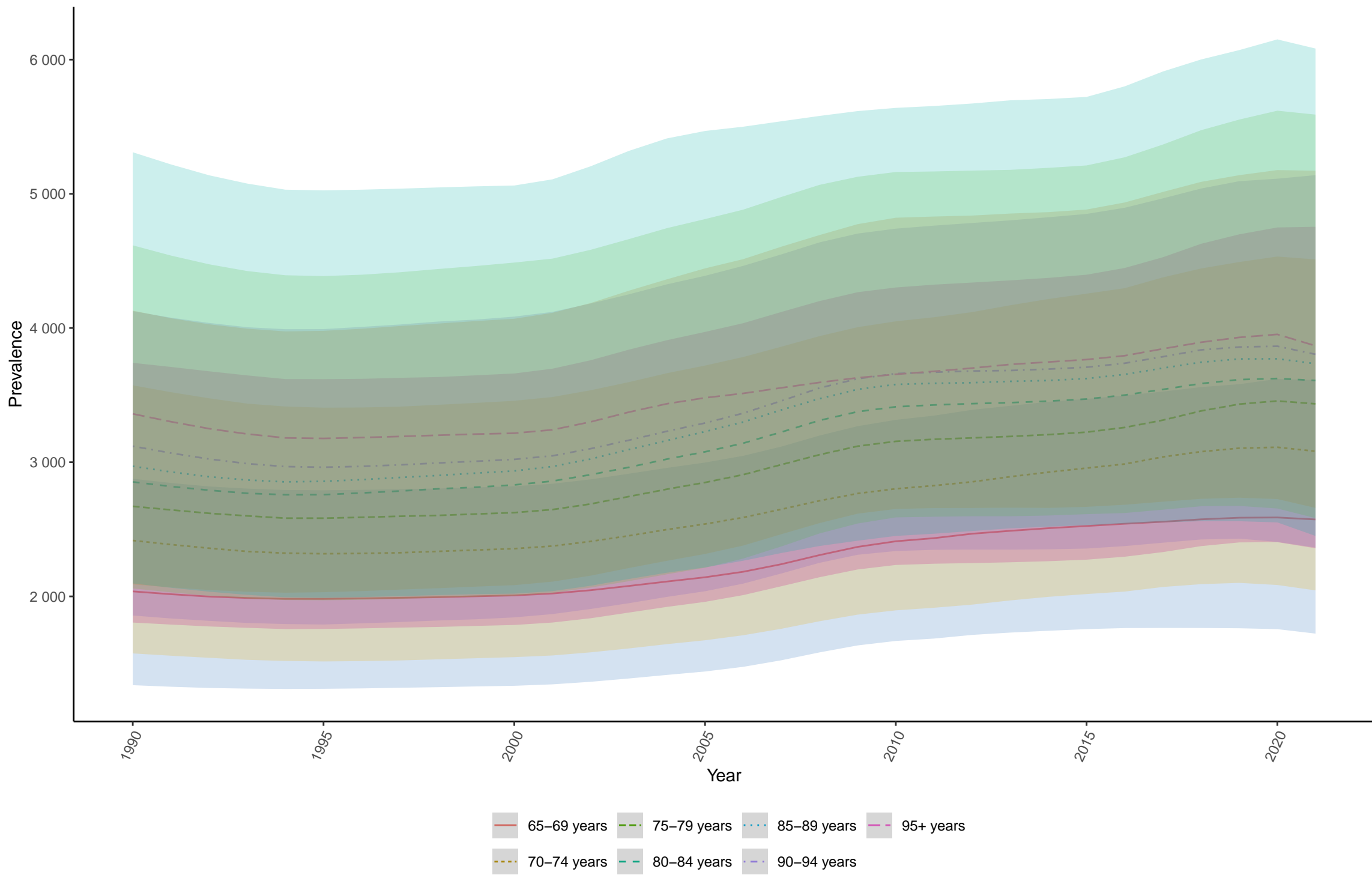

Supplement: Supplementary file 1 [file Data_Sheet_1.zip › Supplementary documents/Supplementary Figure 2∩╝ÜPrevalence-Trends in prevalence over time for different age groups.pdf]

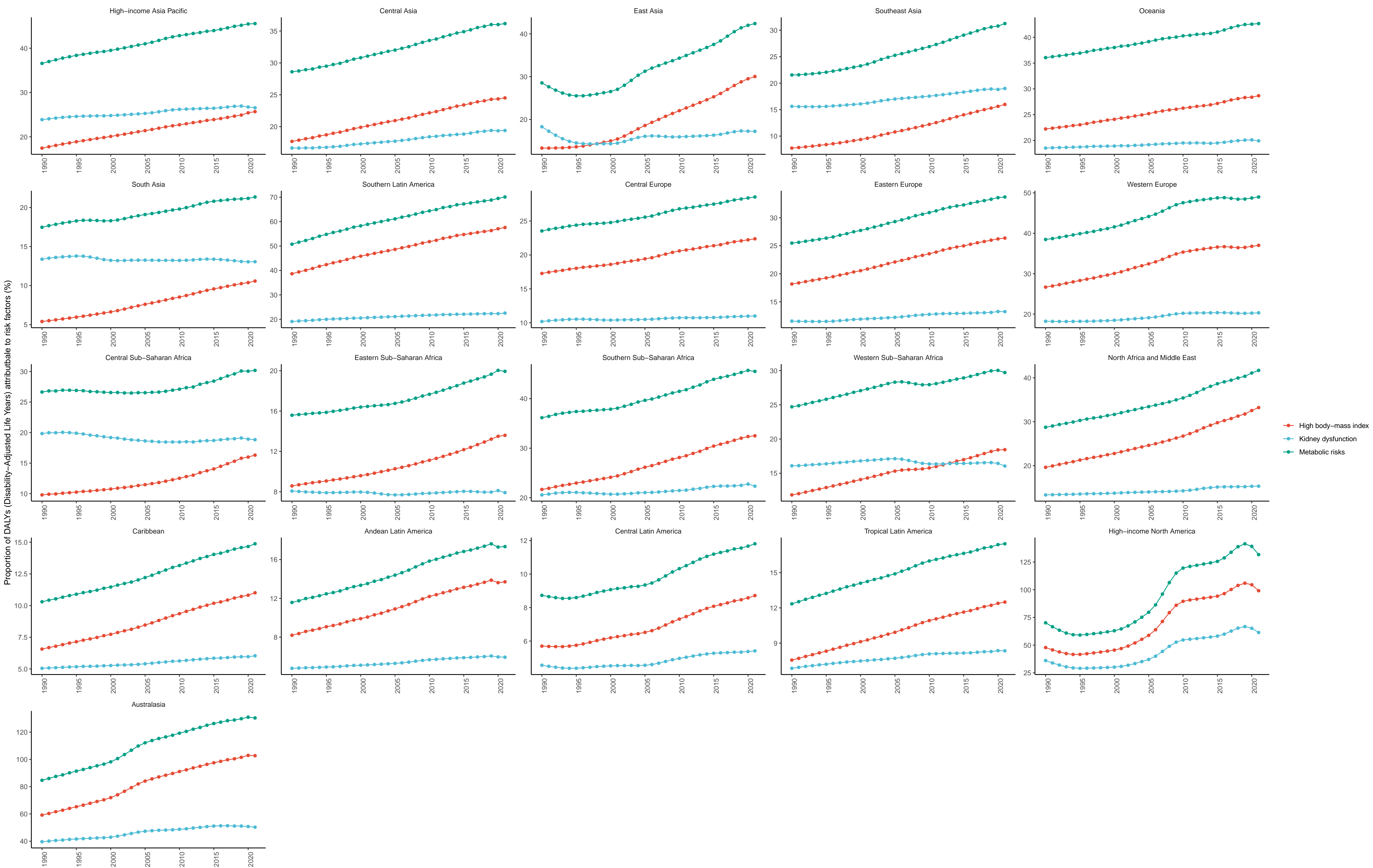

Supplement: Supplementary file 1 [file Data_Sheet_1.zip › Supplementary documents/Supplementary Figure 11∩╝ÜTrends in risk factors for DALYs over time in 21 districts.pdf]

High body-mass index

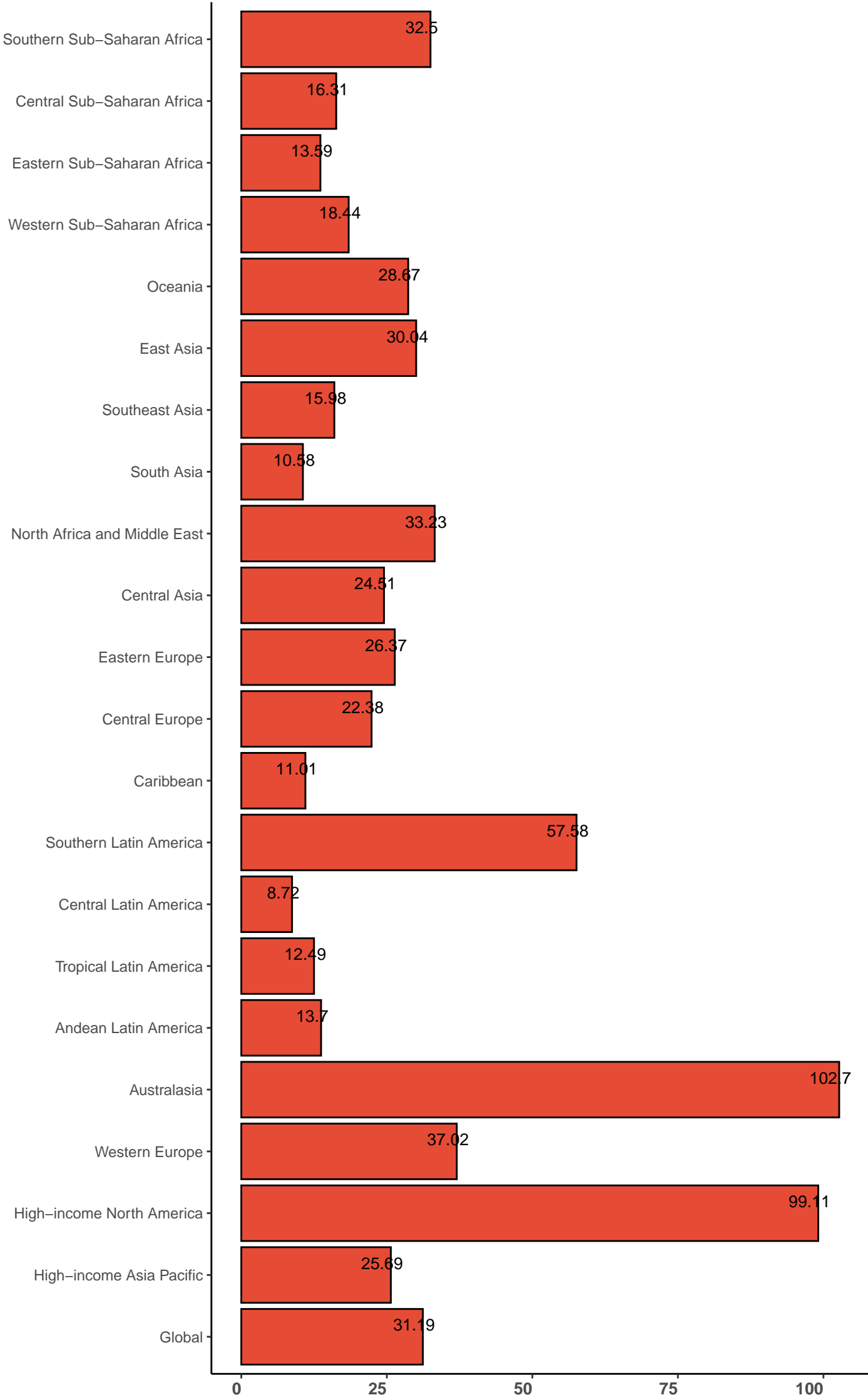

Kidney dysfunction

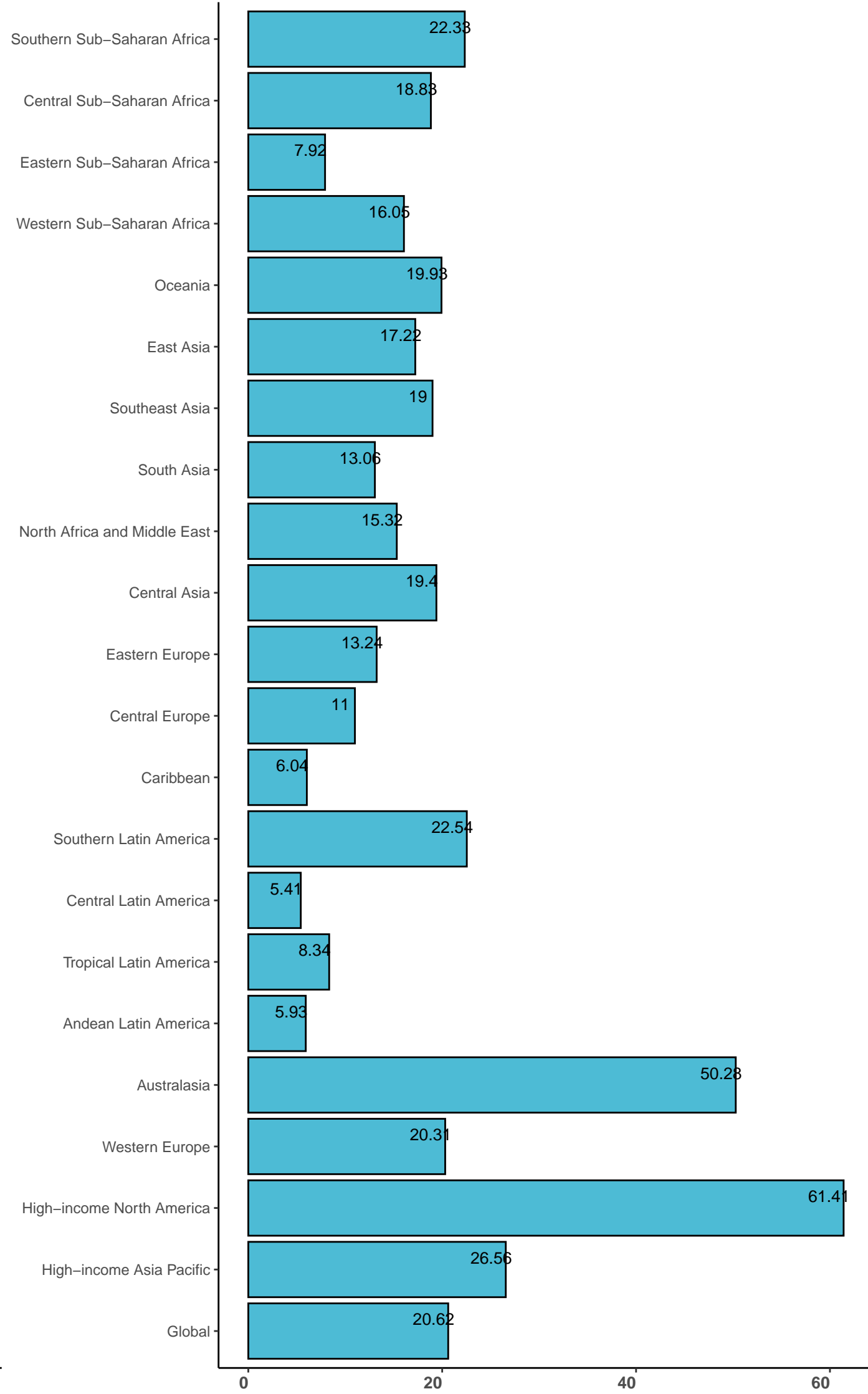

Metabolic risks

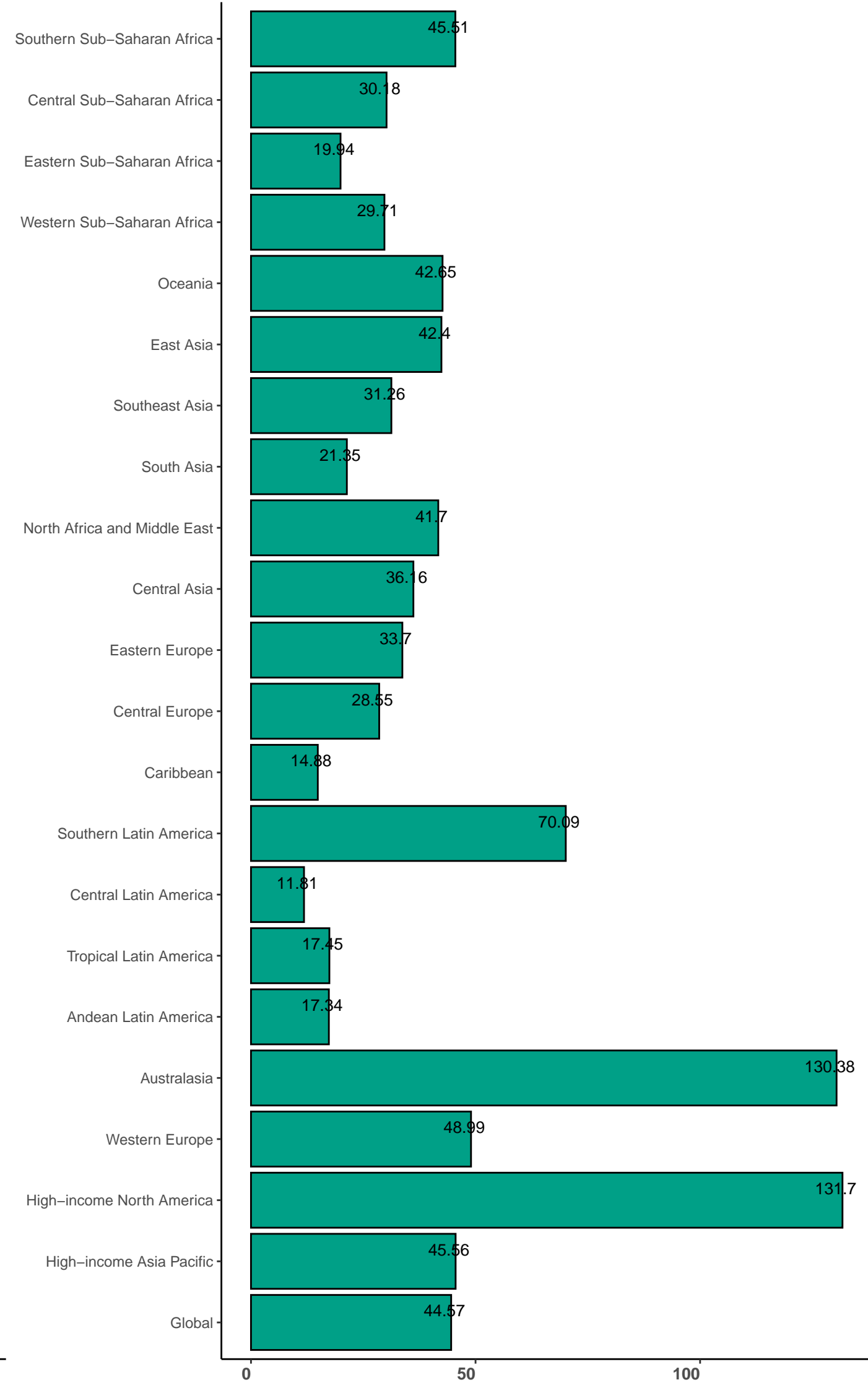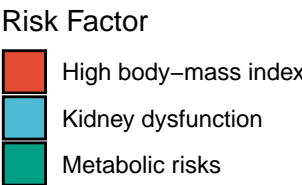

Supplement: Supplementary file 1 [file Data_Sheet_1.zip › Supplementary documents/Supplementary Figure 10Risk factor shares for DALYs in 21 regions (2021).pdf]
